# GATHeR: graph-based accurate tool for immunoglobulin heavy- and light-chain reconstruction

**DOI:** 10.1038/s41467-026-74272-w

**Published:** 2026-06-10

**Authors:** Seyedmojtaba Seyedraoufi, Mari Bergstøl Gornitzka, Andreas Lossius

**Affiliations:** 1 Department of Molecular Medicine, Institute of Basic Medical Sciences, University of Oslo, Oslo, Norway; 2 Department of Neurology, Akershus University Hospital, Lørenskog, Norway

**Keywords:** Antibodies, Immunogenetics, Genome assembly algorithms, B-cell receptor

## Abstract

Recovering full-length, paired B-cell receptor (BCR) sequences from scRNA-seq reads remains difficult, especially in naive and memory B cells where immunoglobulin transcripts are sparse. Incomplete constant-region coverage in current methods limits isoform, subclass, and allele resolution. Here we present GATHeR, an open-source tool that assembles and annotates paired heavy- and light-chain BCR sequences and extends assembled sequences into constant regions. This enables confident subclass and allele assignment and recovery of membrane-bound isoforms, including the transmembrane segment and cytoplasmic tail, thereby distinguishing surface BCRs from secreted antibodies. GATHeR supports Smart-seq2/3 and 10x Genomics libraries and outperforms existing methods across benchmarks, with the largest gains in naive and memory B cells. Notably, in these populations the constant-region extension also enables detection of splice variation, including intron-containing heavy-chain transcripts with read-level support. By delivering high-fidelity receptor, isoform, and clonal lineage information, GATHeR broadens the analytical reach of scRNA-seq for B-cell immunology.

## Abstract (web summary)

Single cell RNA sequencing expanded the possibilities of capturing immune repertoires in lymphocytes but assembling long sequences from the individual reads, especially in naive and memory B cells with low expression of immunoglobulin transcripts is challenging. Here the authors present a computational tool to assemble and annotate paired heavy- and light-chain B-cell receptor sequences which allows distinction of notoriously hard-to-capture splice variants and membrane-bound isoforms.

## Introduction

Humoral immunity depends on antigen recognition by the B-cell receptor (BCR), which comprises a membrane immunoglobulin (Ig)—a heterotetramer of two identical heavy and two identical light chains with variable (V) and constant (C) regions—associated with the CD79a/CD79b (Ig*α*/Ig*β*) signaling heterodimer. Following BCR engagement by cognate antigen and receipt of T cell help, naive B cells either generate short-lived extrafollicular plasmablasts or seed germinal centers for affinity maturation and selection, ultimately producing long-lived plasma cells and memory B cells^1^. Commitment to antibody-secreting fates entails extensive remodeling of protein synthesis and secretory pathways. Accordingly, Ig transcripts can account for up to 70% of total mRNA in antibody-secreting cells versus  approximately 2% in naive or memory populations^2^. This transition is accompanied by regulated alternative splicing and polyadenylation of Ig heavy-chain pre-mRNA at the ${3}^{{\prime} }$ end, which selects the secretory tailpiece rather than the membrane exons (M1/M2) and thus produces the secreted Ig isoform^3^.

Single-cell RNA sequencing (scRNA-seq) now enables joint profiling of cell states and receptor usage. For B cells, accurately recovering paired heavy- and light-chain sequences together with the transcriptome is essential for functional annotation and clonal tracing. To achieve this, standard droplet-based workflows such as 10x Genomics require researchers to prepare additional receptor-specific libraries to capture BCR sequences^4^. Because these assays amplify from CH1-anchored primers, determining Ig subclasses and alleles can be difficult—often impossible. Meanwhile, several full-length scRNA-seq protocols (e.g., Smart-seq2/3^5,6^ and FLASH-seq^7^) have been developed, but in these datasets, BCR transcripts are effectively buried within the complete transcriptome and must be extracted and correctly assembled.

Importantly, most current computational approaches for BCR reconstruction from scRNA-seq are optimized for recovering the V(D)J region and establishing clonotypes. While this is sufficient for many repertoire-level analyses, it is not equivalent to reconstructing full-length heavy- and light-chain transcripts. Achieving constant-region completeness from standard scRNA-seq is challenging because informative constant-region reads can be sparse and discontinuous, especially when overall Ig expression is low (e.g., naive and memory B cells), and because biologically relevant 3′-end processing generates isoforms (membrane-bound versus secreted) that differ in the constant-region tail. Consequently, constant-region reconstruction is often treated as secondary, yet it is essential for robust subclass/isotype and allele assignment and for interrogation of constant-region isoforms and splice variation.

Here we present GATHeR, an open-source pipeline that reconstructs paired, full-length BCRs directly from scRNA-seq. GATHeR supports full-length protocols such as Smart-seq2/3 and also assembles BCRs from 10x Genomics ${5}^{{\prime} }$ gene expression libraries without additional receptor-specific enrichment. Across diverse datasets, GATHeR yields longer, more contiguous BCR assemblies and higher assembly success rates than existing methods, with the largest gains in naive and memory B cells where Ig transcripts are sparse. By extending assemblies across the constant region, GATHeR enables interrogation of constant-region isoforms, subclasses, and alleles, and also reveals splice variation in naive and memory B cells. These capabilities broaden the use of scRNA-seq for detailed, sequence-resolved analyses of B-cell clonality and differentiation.

## Results

### GATHeR assembles and annotates full-length BCRs and supports phylogenetic analysis

**Fig. 1:**
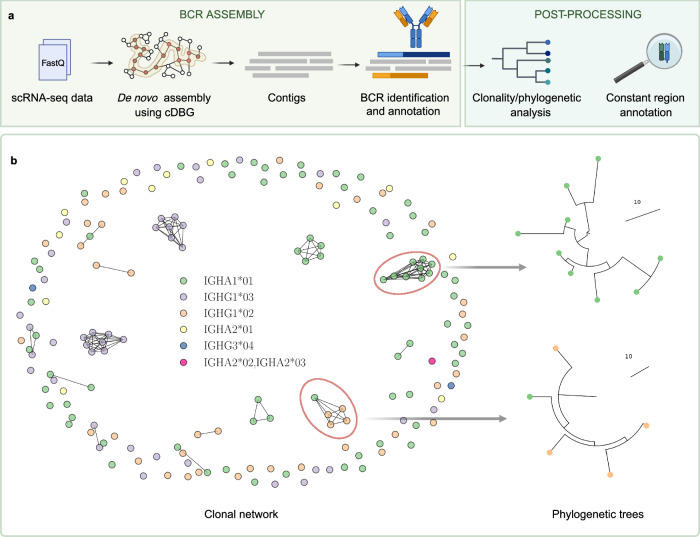
GATHeR reconstructs paired full-length BCRs from scRNA-seq and enables clonality and lineage analysis. **a** Overview of the GATHeR workflow. Created in BioRender. Lossius, A. (2026) https://BioRender.com/r6zb52z. **b** Clonal network of B cells derived from Ig clonality analysis of dataset I in Table 1 (174 human plasmablasts)^9^. Nodes are colored according to Ig isotype. Phylogenetic trees for two highlighted clones are shown on the right.

GATHeR starts by building a compacted de Bruijn graph (cDBG) from scRNA-seq reads (Fig. 1a). Traversing cDBG subgraphs enables recovery of contigs that contain complete Ig transcripts, including the transmembrane and cytoplasmic-tail exons of membrane-bound IgM and IgD in naive B cells. Candidate heavy- and light-chain contigs are identified by alignment to the IMGT germline database, annotated with V(D)J gene usage, constant-region subclass/allele, and contig weight (derived from k-mer frequencies), which is recorded in the FASTA header; secondary contigs are retained whenever multiple heavy or light chains are detected.

A post-processing module wraps Immcantation tools to cluster BCR contigs into clonal families and reconstruct maximum-parsimony lineage trees (Fig. 1b), providing a seamless path from raw reads to repertoire-scale phylogenetic analysis. Importantly, GATHeR annotates all constant-region exons against the IMGT reference, and is able to infer novel constant region alleles (Supplementary Table 1).

### GATHeR achieves high reconstruction yield and sequence-level concordance with existing methods

To evaluate GATHeR, we assembled a primary benchmarking panel comprising four publicly available scRNA-seq datasets spanning plate-based Smart-seq2/3 and droplet-based 10x Genomics ${5}^{{\prime} }$ gene-expression libraries (Datasets I–IV; Table 1). We benchmarked GATHeR against BCR assembly tools that performed well in a recent comparative study^8^: BASIC^9^, BraCeR^10^, BALDR^11^, and TRUST4^12^. BALDR is primarily designed for plate-based Illumina scRNA-seq data from human (and rhesus macaque) B cells and does not provide official support for mouse data; we therefore report BALDR results only for the applicable datasets and exclude it from Dataset II.

**Table 1:** Primary scRNA-seq datasets used for benchmarking GATHeR

Dataset	Platform	Species	Cell type(s)	Cell count	Reference
I^a^	Smart-seq2 + Sanger	Human	Plasmablasts	174	Canzar et al.^9^
II^b^	Smart-seq2	Mouse	IL4/LPS-stimulated B cells	56	Wu et al.^13^
III^c^	Smart-seq3xpress	Human	Naive and memory B cells (1:1)	400	Hagemann-Jensen et al.^6^
IV	10 × Genomics ${5}^{{\prime} }$	Human	Naive and memory B cells, plasmablasts	1111	10x Genomics^48^

^a^Dataset I: paired-end subset used.

^b^Dataset II: batch 1 subset from E-MTAB-4825 (publicly released FASTQs; batch used in BraCeR validation).

^c^Dataset III: 1:1 random sampling of naive and memory B cells.

Dataset I is the original validation dataset for BASIC^9^ and includes Sanger-derived BCR sequences from the same cells, enabling orthogonal validation at both the gene and sequence levels. Dataset II was originally published by Wu et al.^13^ and subsequently used for validation in the BraCeR study^10^. Datasets I–II consist exclusively of antibody-secreting cells and have high immunoglobulin transcript abundance. We therefore additionally included Dataset III, comprising 200 naive and 200 memory B cells sampled at a 1:1 ratio, to evaluate performance when Ig transcripts are sparse. Finally, to assess compatibility with droplet-based 10x Genomics ${5}^{{\prime} }$ gene-expression libraries lacking receptor-specific enrichment, we included Dataset IV, previously used to validate TRUST4^12^.

Across Datasets I-II, all evaluated tools recovered paired heavy-light chains for almost all cells (Supplementary Fig. 1a), noting that BALDR was not run on Dataset II. In Dataset III (naive and memory B cells), GATHeR reconstructed BCRs for 399/400 cells (one heavy chain missing) and TRUST4 for 398/400 (two heavy chains missing). BASIC recovered BCRs for 94% of cells (23 heavy and 24 light chains missing). BraCeR recovered heavy chains for 78% of cells (312/400) and light chains for 96% (383/400), while BALDR recovered heavy and light chains for 72% (287/400) and 71% (285/400), respectively. Among reconstructed contigs, sequence-level concordance with GATHeR was high across all datasets (Supplementary Fig. 1b, c). BraCeR showed 98–100% of contigs with similarity ≥0.9, and TRUST4 93–98% (heavy) and 91–98% (light). BASIC was lower in Datasets I-II (64–84% heavy; 66–79% light) but reached ~99.7% in Dataset III. Sequences recovered by BALDR were also highly similar to GATHeR (Dataset I: 96% heavy, 95% light; Dataset III: 96% heavy, 96% light).

### GATHeR reconstructs longer, more complete BCRs with extended constant-region coverage

**Fig. 2:**
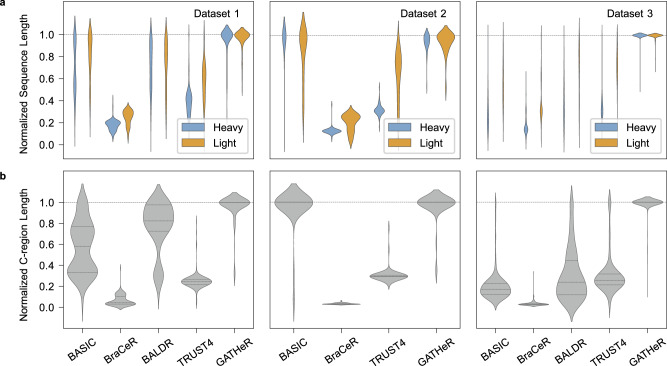
GATHeR reconstructs longer, more complete heavy- and light chains across datasets. **a** Distributions of contig lengths for heavy- and light-chain assemblies produced by GATHeR and benchmark methods (BASIC, BraCeR, BALDR, TRUST4) across Datasets I-III (BALDR not evaluated on the mouse dataset, Dataset II). For each cell and chain type, lengths are normalized to the longest contig produced by any method for that cell (so 1 denotes the longest per-cell contig among methods). **b** Corresponding heavy-chain constant-region coverage, shown as normalized length distributions using the same per-cell normalization.

Long, contiguous BCR sequences spanning the variable region and the full constant region of both heavy and light chains enable confident isotype/subclass and allele assignment and support robust clonal lineage analysis. Across Datasets I–III, GATHeR typically produced near full-length heavy- and light-chain sequences (median normalized length ≈ 1.0; Fig. 2a). Although variability was high, some BASIC and BALDR reconstructions in Dataset I reached lengths comparable to GATHeR, and BASIC produced near full-length sequences in Dataset II; in contrast, both BASIC and BALDR yielded consistently shorter sequences in Dataset III. BraCeR and TRUST4 produced shorter reconstructions across all datasets. Heavy-chain constant-region coverage (Fig. 2b) mirrored these trends, with GATHeR recovering the longest constant-region segments across datasets.

### GATHeR matches Sanger-derived ground truth and remains accurate under increasing somatic hypermutation (SHM)

To establish nucleotide-level correctness beyond cross-tool concordance, we performed orthogonal validation against matched Sanger-derived BCR sequences from two independent plate-based datasets (Dataset I and an additional Smart-seq2 memory B-cell dataset with Sanger ground truth; Supplementary Table 2). We quantified gene-level agreement (V, D, J and, where applicable, constant-region/isotype assignment), sequence-level concordance (local identity and truth coverage) and error signatures, including INDEL rates (indels per kb from V-segment alignments) and a ground-truth discordance rate (V(D)J-set discordance), used as a proxy for misassemblies (“Methods”). In Dataset I, some Sanger sequences include vector-derived constant regions following cloning and therefore support V(D)J validation but not constant-region validation, motivating the inclusion of the independent memory B-cell dataset for full-length (including constant-region) ground truth.

**Fig. 3:**
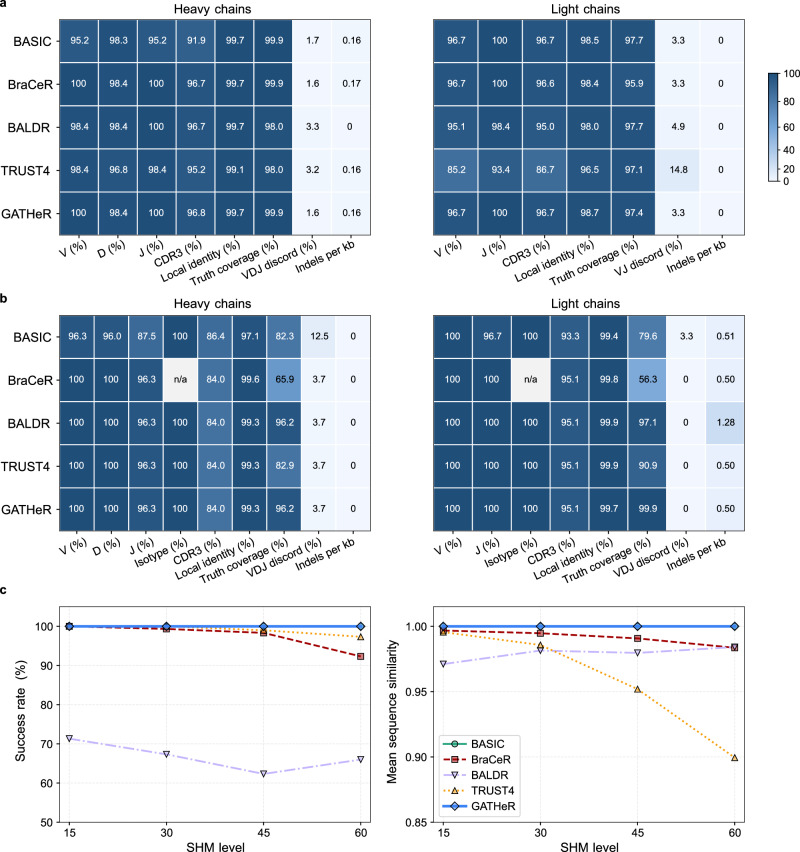
GATHeR matches Sanger-derived ground truth and remains robust under increasing somatic mutations. **a** Dataset I: V/D/J agreement, sequence concordance (local identity, truth coverage), and error signatures (INDEL rate; V(D)J-set discordance as a proxy for major incorrect reconstructions). **b** Independent Smart-seq2 memory B-cell dataset: same metrics as in (a), plus constant-region/isotype agreement where covered by Sanger. **c** Somatic hypermutation (SHM) stress test on in silico Illumina reads simulated from templates (100 IGH, 100 IGK, 100 IGL) with 15/30/45/60 substitutions per sequence. *n/a*: not evaluable by our IgBLAST-based constant-region calling (contig too short).

In Dataset I, all methods showed high gene- and sequence-level agreement to the Sanger truth for both heavy and light chains, with low INDEL rates and low V(D)J-set discordances (Fig. 3a). For heavy chains, GATHeR achieved 100% V and J agreement (D: 98.4%) with high sequence concordance (local identity 99.7%, truth coverage 99.9%), and low error signatures (VDJ-set discordance 1.6%; 0.16 indels per kb). For light chains, GATHeR similarly showed high gene-level agreement (V: 96.7%, J: 100%) and high sequence concordance (local identity 98.7%, truth coverage 97.4%), with low VJ-set discordance (3.3%) and no INDEL evidence in this dataset (0 indels per kb).

In the independent memory B-cell dataset, where Sanger ground truth sequences include native constant-region sequence, the same evaluation additionally enabled constant-region/isotype agreement (Fig. 3b). Here, GATHeR recovered substantially more of the Sanger-supported sequence span than comparator methods, most prominently for light chains (truth coverage 99.9% for GATHeR versus 90.9% for TRUST4, 97.1% for BALDR, 56.3% for BraCeR, and 79.6% for BASIC) and also for heavy chains (96.2% for GATHeR versus 82.9% for TRUST4, 96.2% for BALDR, 65.9% for BraCeR, and 82.3% for BASIC), while maintaining low V(D)J-set discordances (3.7% heavy; 0% light) and low INDEL rates (0 indels per kb heavy; 0.50 indels per kb light). Local identity was comparable across methods in this dataset. Together, these orthogonal validations support accurate BCR reconstruction by GATHeR at nucleotide resolution.

To probe robustness under high sequence divergence, we next evaluated performance under controlled increases in SHM using an in silico Illumina dataset derived from published BCR template sequences (100 IGH, 100 IGK, and 100 IGL) and spanning 15, 30, 45, or 60 substitutions per sequence^8^. Across chains, GATHeR maintained high assembly success and near-constant similarity as SHM levels increased (Fig. 3c), whereas comparator methods showed reduced success and/or declining similarity at higher mutation rates, most prominently for TRUST4 at the highest mutation burdens. These results complement the Sanger-based analyses and support robust performance under substantial SHM.

### Full-length constant-region extension resolves membrane-bound isoforms and identifies candidate splice variation in naive and memory B cells

**Fig. 4:**
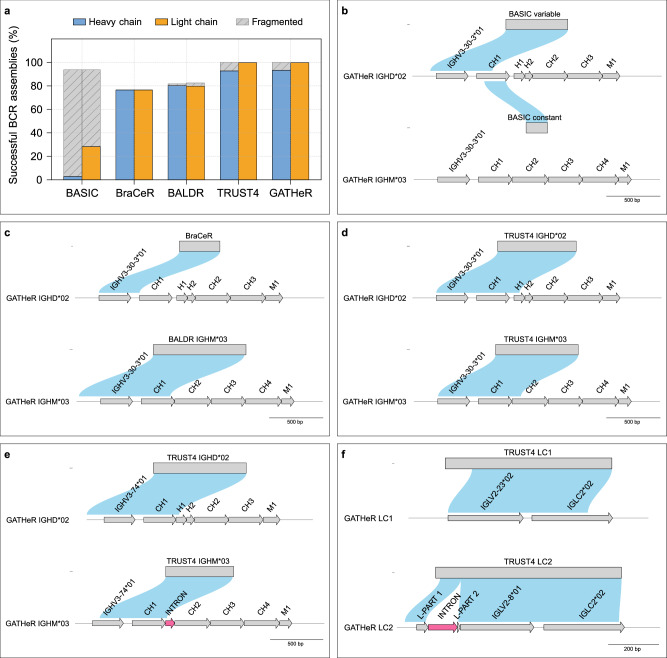
GATHeR preserves sequence contiguity and identifies candidate intron-containing regions in BCR assemblies. **a** Percentage of cells with contiguous heavy- and light-chain assemblies for each method in Dataset III (naive and memory B cells); the fraction of fragmented assemblies is shown in gray. **b–d** Representative naive B cell: GATHeR-assembled IgM and IgD heavy-chain contigs aligned with assemblies from other methods: **b** BASIC, **c** BraCeR and BALDR, and **d** TRUST4. **e** Example of a candidate intron-containing region in the IgM constant region detected in a GATHeR contig; the aligned TRUST4 reconstruction does not include this region. **f** Light-chain example showing a candidate partial intron between L-PART1 and L-PART2 in a GATHeR contig; the aligned TRUST4 reconstruction does not include this region.

Because naive and memory B cells represent a particularly challenging setting due to sparse Ig transcripts, we next focused on Dataset III to assess assembly integrity and to examine constant-region features enabled by full-length reconstruction. Sequence integrity differed markedly among methods (Fig. 4a). GATHeR delivered the highest contiguity, assembling heavy- and light-chain sequences as contiguous in 93% and 100% of cells, respectively. By contrast, BASIC achieved contiguity in only 9% (heavy chains) and 35% (light chains) of cells; TRUST4 closely matched GATHeR, with marginally lower contiguity for heavy chains, while BraCeR produced mostly contiguous heavy and light chains but at a lower overall success rate.

Mature naive B cells are known to co-express IgM and IgD^14^. In Dataset III, GATHeR reconstructed both IGHM- and IGHD-assigned heavy-chain contigs in 88% of annotated naive B cells; among these, 77% shared an identical CDR3 nucleotide sequence, consistent with IgM/IgD constant-region isoform co-expression from a single IGH VDJ rearrangement (Supplementary Fig. 2a). Panels b–d of Fig. 4 illustrate BCR reconstruction in a representative naive B cell: GATHeR reconstructs two full-length heavy-chain isoforms (IgM and IgD), whereas BASIC (Fig. 4b) splits one isoform into variable- and constant-region fragments. BraCeR and BALDR (Fig. 4c) each partially reconstruct one isoform, and TRUST4 (Fig. 4d) returns shorter reconstructions of both isoforms.

In Dataset III, GATHeR also reported multiple reconstructed IGH contigs in a subset of annotated memory B cells (Supplementary Fig. 2a–c). Importantly, these multi-IGH cases were dominated by IGHM/IGHD contigs sharing the same V region (identical CDR3 nucleotide sequence; Supplementary Fig. 2a), consistent with unswitched memory or naive-like phenotypes rather than two independent productive receptors. Accordingly, when separating isoform pairs (same CDR3) from truly distinct IGH rearrangements (different CDR3), only approximately 2% of memory B cells contained two distinct IGH rearrangements (Supplementary Fig. 2b). Multiple light-chain contigs were also observed in a subset of cells (Supplementary Fig. 2d), and productive versus non-productive classifications for additional heavy- and light-chain contigs are summarized in Supplementary Fig. 2e, f.

GATHeR’s extended heavy- and light-chain contigs enabled detection of splicing-related features beyond V(D)J reconstruction. In Dataset III, we detected candidate intron-containing heavy-chain transcripts in 8% of naive and 9% of memory B cells, and in light chains in 10% and 23% of cells, respectively. Graphical representations of the heavy- and light-chain splice forms are shown in Supplementary Figs. 3 and 4, respectively. Among these intron-containing sequences, 37/38 heavy chains (97%) and 77/79 light chains (97%) contained a frameshift and/or a premature termination codon, and were therefore classified as non-productive (Supplementary Fig. 2c, d). To assess assembly-independent support, we mapped the reads from the corresponding cells to the GATHeR contigs and quantified coverage over annotated exon and intron intervals (Supplementary Fig. 5). All intron intervals showed read support, with lower mean depth over introns than exons (Supplementary Fig. 5a, b). We additionally observed reads spanning exon–intron junctions (Supplementary Fig. 5c), and sashimi plots showing junction-spanning support consistent with both spliced and intron-containing transcripts across multiple isotypes (Supplementary Fig. 6). Figure 4e shows an example of an intron-containing region in the constant region of a GATHeR-reconstructed IgM transcript from a naive B cell; the corresponding TRUST4 reconstruction does not capture this region and would classify the transcript as productive. A representative light-chain example is shown in Fig. 4f: GATHeR reconstructs a light chain containing a partial intron between the first and second leader exons (L-PART1 and L-PART2), introducing a premature termination codon. These observations are consistent with prior reports of light-chain intron retention in naive B cells^15^ and extend the set of possible intron-containing transcripts to include heavy chains in both naive and memory B cells.

### GATHeR successfully assembles BCRs from 10x Genomics ${5}^{{\prime} }$ gene expression libraries

**Fig. 5:**
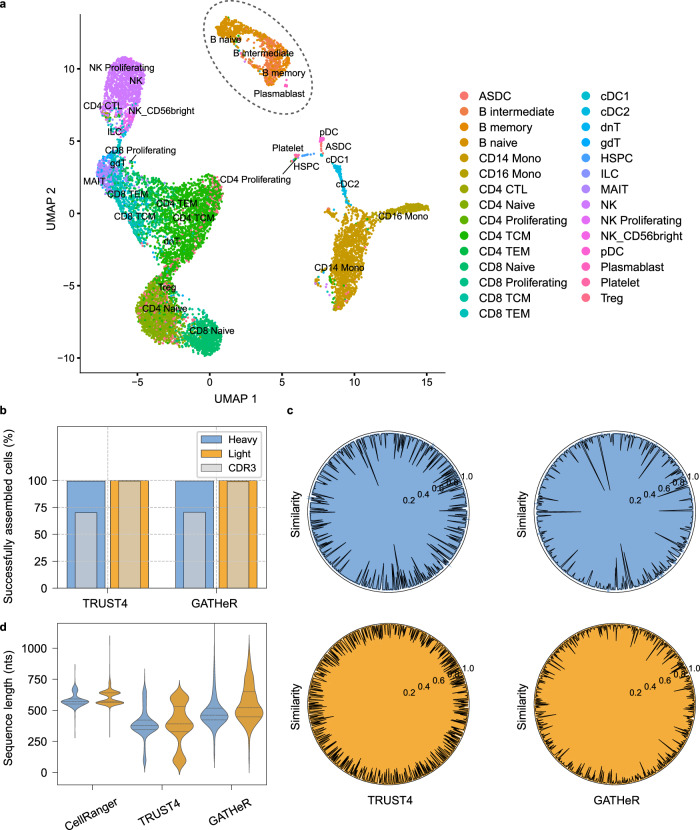
GATHeR assembles accurate BCRs from 10x Genomics ${5}^{{\prime} }$ gene expression libraries with high concordance to 10x V(D)J references. **a** UMAP of PBMCs computed and annotated in Seurat. **b** Percentage of annotated B cells in which GATHeR and TRUST4 assembled heavy chains, light chains, and sequences with complete CDR3 regions. **c** Radar plots showing length-normalized nucleotide identity of heavy- and light-chain assemblies relative to the 10x Genomics V(D)J reference (identical nucleotides divided by the length of the shorter sequence). **d** Nucleotide length of assembled BCR sequences compared with the 10x Genomics V(D)J reference.

Unlike the standard 10x Genomics workflow, which typically relies on an additional V(D)J-enriched library, GATHeR reconstructs BCRs directly from ${5}^{{\prime} }$ gene-expression (GEX) data, eliminating that step. Because TRUST4 is the only comparator with native GEX support, we benchmarked GATHeR against TRUST4 on 1,111 Seurat-annotated naive and memory B cells together with a small proportion of plasmablasts (Fig. 5a; Table 1, Dataset IV). Both methods assembled at least one chain in > 99% of cells (Fig. 5b). For sequences spanning CDR3, GATHeR reconstructed heavy and light chains in 70.5% and 99.0% of cells, respectively (TRUST4: 70.1% and 99.5%).

Accuracy was assessed against annotations derived from the matched 10x Genomics V(D)J-enriched library (high-confidence reference; Fig. 5c). Both tools were concordant with the benchmark, with GATHeR yielding higher mean similarity (heavy: 0.97 vs. 0.95; light: 0.98 vs. 0.95). Among barcodes with both productive and additional non-productive IGH calls in the matched V(D)J reference, GATHeR recovered all productive chains and 9/13 additional non-productive IGH contigs from GEX in this subset (Supplementary Table 3). In addition, GATHeR produced longer reconstructed sequences than TRUST4 (Fig. 5d), in some cases approaching lengths obtained from the pre-amplified V(D)J library.

Taken together, these results indicate that GATHeR delivers both longer and more accurate BCR reconstructions from 10x Genomics gene-expression data than TRUST4, without the need for an additional enrichment library.

## Discussion

GATHeR consistently reconstructs longer, more contiguous BCR assemblies than existing methods, with the largest gains in naive and memory B cells where Ig transcripts are sparse. By recovering the entire constant regions of both heavy and light chains, GATHeR enables reliable isotype/subclass and constant-region allele assignment, strengthens clonal lineage inference, and allows direct interrogation of membrane (M1/M2) versus secretory tailpiece usage. Extended assemblies also enable detection of candidate intron-containing regions in a measurable fraction of heavy- and light-chain transcripts, frequently introducing premature stop codons and resulting in non-productive sequences. Such splice-variant–like features are not routinely captured by V(D)J-focused reconstruction workflows and can be missed when the constant-region sequence is incomplete or fragmented. Collectively, these results position GATHeR as a robust, end-to-end solution for sequence-resolved BCR analysis across library types and cellular states.

Although BCR diversity is often equated with V(D)J variation, human constant regions harbor substantial genetic and structural diversity, including population- and subclass-specific polymorphisms and hinge-length variants, beyond what is typically captured in repertoire studies^16–18^. Changes in heavy-chain constant domains can tune effector mechanisms by altering C1q engagement and Fc*γ*R interactions (affecting complement activation, ADCC and ADCP)^19–21^, and variants that modulate FcRn binding can influence antibody transport and half-life^22^. Substitutions in CH1—and in some contexts CH2/CH3—can allosterically impact antigen binding by the Fab^23^. At the BCR itself, a cytoplasmic-tail variant (G396R) associates with altered B-cell activation, lupus risk and vaccine responses^24^. By recovering complete constant regions alongside the V(D)J, GATHeR enables reliable sub-isotype and constant-region allele calls and a direct readout of membrane versus secretory isoforms, providing a sequence-resolved framework to connect constant-region genotype with cellular function.

GATHeR’s full-length immunoglobulin contigs enable detection of candidate intron-containing heavy-chain transcripts that can render BCR sequences non-productive. Unproductive splicing is conserved across species^25^ and intron retention has long been considered an additional layer of gene-expression control^26^, yet the underlying machinery and regulatory logic remain incompletely understood. Partial intron retention has been reported in the ${5}^{{\prime} }$ UTR of *κ*- and *λ*-light-chain transcripts in naive human B cells^15^. Here, we observe similar intron-containing light-chain transcripts and extend these observations to heavy chains in both naive and memory B cells. Because single-cell RNA-seq may capture traces of unspliced nuclear pre-mRNA, we cannot conclusively distinguish bona fide intron retention from pre-mRNA in all cases without orthogonal validation. Nevertheless, the presence of partially retained introns and the accompanying read-level support across multiple cells are consistent with splice-variant–like events rather than solely background pre-mRNA leakage. Notably, many of these intron-containing heavy-chain transcripts involve distal (${3}^{{\prime} }$) constant region—segments (e.g., near the membrane-coding exons), highlighting the value of full-length constant-region reconstruction and offering one explanation for why V(D)J-focused approaches may miss such features.

GATHeR reconstructed two or more immunoglobulin contigs (heavy- and/or light-chain) in a subset of B cells, consistent with prior single-cell reports^27,28^. In naive B cells, the dominant multi-IGH pattern comprised paired IGHM and IGHD contigs sharing the same VDJ junction (identical CDR3 nucleotide sequence), consistent with the canonical model in which a single rearranged VDJ exon is expressed with alternative constant regions. This highlights an advantage of full-length reconstruction for resolving constant-region isoform diversity, which can be difficult to capture with approaches that do not reliably span the full constant region. Multi-contig reconstructions were also observed in some memory B cells; however, separating IGHM/IGHD isoform pairs (same CDR3) from truly distinct IGH rearrangements (different CDR3) shows that the multi-IGH signal is dominated by isoform pairs consistent with unswitched memory phenotypes^29^, whereas distinct dual-IGH rearrangements are rare (Supplementary Fig. 2b). Accordingly, we interpret multi-contig output conservatively: additional contigs may reflect constant-region isoforms, non-productive rearrangement history, and/or low-support secondary reconstructions. Barcodes containing multiple productive contigs with discordant VDJ junctions can also reflect technical factors (e.g., multiplets or ambient RNA), and without orthogonal validation, these events should not be used to support biological conclusions. In line with standard practice in single-cell BCR reconstruction workflows, we treat multi-contig barcodes cautiously and avoid over-interpreting them as evidence of co-expression of multiple functional receptors.

Methodologically, GATHeR builds on a long line of assembly strategies grounded in de Bruijn graphs (DBGs). DBGs have powered short-read assembly since the Eulerian-path formulation^30^ and tools like Velvet^31^; Trinity adapted DBGs to transcriptomics for isoform-aware de novo assembly^32^. In BCR reconstruction, BraCeR and BALDR apply Trinity after per-cell Ig read enrichment—preserving isoforms and SHM but at a higher compute cost^10,11^. By contrast, BASIC stitches from germline anchors, TRUST4 uses k-mer seeding with greedy extension, and MiXCR is alignment-guided^9,12,33^. GATHeR avoids upfront alignment/filtering and combines (i) a compacted DBG with lightweight path traversal for well-covered loci and (ii) rnaSPAdes assembly algorithm^34^, which has demonstrated advantages over Trinity in producing more complete assemblies with fewer chimeras and less redundancy, while handling uneven or low coverage more effectively—capabilities that are critical for naive and memory B cells where sparse coverage and co-expression of multiple heavy chain isotypes (e.g., IgM and IgD) are common. In our ground-truth validation, GATHeR’s isotype-level assignment was comparable to other methods when the constant-region sequence was sufficiently covered (Fig. 3). However, GATHeR’s added value is more complete and contiguous constant-region reconstruction, enabling subclass/allele calls and isoform resolution. Consistent with this design, our controlled SHM stress test with known ground truth indicates stable reconstruction performance under increasing substitution burdens, supporting robustness under high divergence from germline reference sequences—a practical failure mode for pipelines that rely on germline-anchored read selection or seeding. A current limitation is that GATHeR targets immunoglobulin loci (BCR) and does not yet support T-cell receptor (TCR) reconstruction. The assembly principles underlying GATHeR can, in principle, be extended to TCR loci, and ongoing work is adapting the framework to TCR-specific locus structure and chain annotation.

In conclusion, GATHeR expands what can be inferred from Ig transcripts in scRNA-seq: when constant-region reads are available, it routinely generates assemblies that span the entire constant region, detects splice-derived nonproductivity and co-expressed isoforms, and operates on both full-length and ${5}^{{\prime} }$ gene-expression libraries. These capabilities extend the possibilities for single-cell analyses of B cells in vaccination, autoimmunity, and malignancies.

## Methods

### Graph construction and assembly strategy

GATHeR employs compacted de Bruijn graphs (cDBGs) to facilitate the de novo assembly of RNA sequence data. In contrast to approaches primarily optimized for V(D)J/CDR3 recovery and clonotyping, GATHeR is designed to prioritize full-length heavy- and light-chain reconstruction with maximal constant-region completeness when supporting reads are present. This objective motivates a graph-guided assembly strategy that favors long, well-supported paths under sparse and uneven coverage and, when needed, incorporates transcriptome-assembly contigs to bridge low-coverage regions. Graph construction uses a default k-mer size of *k* = 25.

The method utilizes BCALM2 (v2.2.3)^35^ to compact the de Bruijn graphs (DBGs), where nodes correspond to the maximal unitigs of the original DBG. This compaction reduces computational cost without losing information. Nodes are weighted based on the average abundance of the compacted *k*-mers, while edges are weighted by the mean weights of the connected nodes. The rnaSPAdes assembler (part of the SPAdes package; v4.2.0)^34^ is invoked when read coverage is low and/or uneven. In such cases, rnaSPAdes-derived contigs are also incorporated into the BCR identification step.

### Error correction and graph simplification

*k*-mer errors are addressed in two steps. The first step involves the compaction process, where *k*-mers with a frequency below a certain threshold are removed. This threshold can be adjusted based on the characteristics of the data. The second step, which also contributes to graph simplification, involves removing duplicate unitigs with lower weights. Let *u* and *v* be two unitigs in a set *S*, and let *w*(*u*) and *w*(*v*) denote their respective weights. *pre*(*u*, *k* − 1) and *suf*(*u*, *k* − 1) are defined as the first and the last *k* − 1 characters of *u* respectively. Then *u* and *v* are duplicates when *pre*(*u*, *k* − 1) = *pre*(*v*, *k* − 1) or *suf*(*u*, *k* − 1) = *suf*(*v*, *k* − 1). The new set S^*^, used to build the cDBG, contains only those *u* that have the highest weights among all satisfying the overlapping condition in *S*. Additionally, errors are implicitly corrected during the traversal and subsequent identification of the optimal path on the graph, which will be explained shortly.

### Traversing and finding optimum paths

To make traversing and pathfinding more efficient, we decompose the cDBG into a set of subgraphs by identifying weakly connected components (WCCs) using NetworkX (v3.2.1)^36^. These components are the maximal subgraphs of the cDBG where an undirected path exists between any pair of nodes. We assume each WCC corresponds to a single contig, and therefore treat each WCC as an independent search space. Accordingly, we employed the depth-first search (DFS) algorithm to explore each subgraph and generate candidate paths for contig recovery. Let subgraph ${G}^{{\prime} }$ be an element of the set of WCCs. Then a candidate path *P* comprises a sequence of nodes *P* = [*u*_1_, *u*_2_, …, *u*_*k*_] generated during the DFS exploration of ${G}^{{\prime} }$. Path *P* is considered optimal if it has the largest component-normalized support score *ϕ*(*P*), which is defined as:

1 $$\phi (P)=\frac{{\sum }_{u\in P}w(u)}{\max (| P| )}$$

where ∣*P*∣ represents the number of nodes in the path *P* and $\max (| P| )$ is the length of the longest path in ${G}^{{\prime} }$. Note that $\max (| P| )$ is constant within each ${G}^{{\prime} }$ and is used only to keep scores on a comparable scale; it does not convert *ϕ*(*P*) into an average per node. By prioritizing high-support, long paths within each component, this scoring scheme favors contiguous reconstructions that are more likely to span into the constant region when sufficient evidence is present, rather than optimizing solely for V(D)J/CDR3 recovery.

### Immunoglobulin sequence identification and contig weight normalization

IMGT references are used for post hoc identification and exon-level annotation of de novo contigs, rather than to constrain assembly to a predefined transcript structure. Assembled contigs were mapped to human and mouse Ig variable and constant region references using local sequence alignment. By default, the method uses BLASTN (NCBI BLAST+ v2.16.0)^37^ with the following parameters for pairwise alignments: Percentage Identity (perc_identity) = 90%, E-value (evalue) < 0.01, and Bit Score (bitscore) > 50. GATHeR then annotates the heavy- and light-chain assemblies and outputs them, along with their corresponding weights, in FASTA format. Within each chain type (heavy vs light), contigs are scored by mean k-mer abundance and converted to a normalized weight to account for score-scale differences between contigs produced by different assembly steps. This normalized weight is reported in the FASTA header and used to rank and report primary and secondary contigs. As part of the post-processing step, GATHeR also uses a built-in aligner based on Biopython (v1.84)^38^ to annotate constant domains of Igs. Genomic alignments and schematic visualizations of reconstructed immunoglobulin contigs shown in Fig. 4 were generated using the Python package pyGenomeViz^39^.

### Clonality and phylogenetic analysis

As a post-processing step, an optional command uses existing tools for clonality analysis and lineage tree construction. Assembled BCR sequences from different single-cell datasets can be collected and processed using IgBLAST (v1.22.0)^40^ and Change-O (v1.3.4)^41^ to comprehensively profile the Ig repertoire. This analysis identifies the most likely germline V, D, and J genes and delineates the junction regions, including the complementarity-determining region 3 (CDR3), for each sequence. Change-O uses these annotations to cluster sequences into clonotypes. Subsequently, Dowser (v1.10.0) and IgPhyML (GitHub-repository version) are employed to construct lineage trees based on clonal assignment information combined with light chain data^42,43^.

### Benchmark dataset selection and inclusion criteria

The primary benchmarking panel comprised four public scRNA-seq datasets (Datasets I–IV; Table 1). Where applicable, we restricted analyses to subsets with available raw FASTQ files and to a uniform paired-end configuration to avoid confounding by sequencing mode. Dataset I contains both paired-end and single-end cells; we therefore analysed the paired-end subset for consistency across benchmarks. For Dataset II (E-MTAB-4825), we analysed the batch 1 cells with available raw FASTQs to ensure full reproducibility from public data. For Dataset III (E-MTAB-11452), we randomly sampled 200 naive and 200 memory B cells (1:1) from the full dataset to create a tractable panel representative of sparse Ig transcript settings; the exact barcode list used for the subsample is provided in Source Data. Dataset IV comprised all Seurat-annotated B cells from the 10x Genomics PBMC dataset used for the gene-expression (GEX) assembly benchmark.

### Orthogonal accuracy evaluation against Sanger-derived ground truth

We evaluated nucleotide-level reconstruction accuracy against matched Sanger-derived BCR sequences in two independent plate-based datasets (Dataset I and an additional Smart-seq2 memory B-cell dataset; Supplementary Table 2). For each method, we compared the best-supported contig per cell to the Sanger truth and quantified gene-level agreement, sequence-level concordance, and error signatures. For gene-level agreement, V, D, and J gene segments (and constant-region/isotype where applicable) were assigned using IgBLAST. Sequence-level concordance was summarized by (i) local identity, defined as the fraction of identical positions among aligned positions, and (ii) truth coverage, defined as the number of identical aligned positions divided by the length of the Sanger ground-truth sequence. Indel errors were quantified from V-segment alignments and reported as indels per kb. To screen for major incorrect reconstructions consistent with potential misassemblies, we additionally reported a ground-truth discordance rate (V(D)J-set discordance), defined as the fraction of reconstructions whose V(D)J gene set did not match the Sanger truth. In Dataset I, some Sanger entries contain vector-derived constant regions following cloning and therefore support V(D)J-level validation but not constant-region validation; constant-region/isotype agreement was therefore evaluated primarily in the independent memory B-cell dataset.

### Generating in silico Illumina data across increasing somatic hypermutation levels

We used the BCR reference sequences published by Andreani et al. as input for read simulation^8^. These data comprise four sets of synthetic immunoglobulin variable-region sequences with increasing levels of somatic hypermutation (15, 30, 45, and 60 SHMs), each set containing 100 heavy-chain (IGH), 100 kappa light-chain (IGK), and 100 lambda light-chain (IGL) sequences. In the original benchmark, the sequences were generated with immuneSIM using a data-driven SHM model that concentrates mutation events in IMGT-defined CDRs^44^, and were designed to stress-test BCR reconstruction methods across SHM regimes. To generate paired-end Illumina reads from these reference sequences, we used ART (ART_Illumina Q) v2.5.8^45^. For each SHM level, we simulated 75-bp paired-end reads (-p -l 75) with a total of 500,000 read pairs (-c 500000). Fragment sizes were drawn from a normal distribution with mean 150 bp and standard deviation 10 bp (-m 150 -s 10). To minimize indel-related artifacts and keep the benchmark focused on substitution-driven divergence from germline (i.e., SHM), we set the insertion rates for read 1 and read 2 to 1 × 10^−5^ (-ir 0.00001 -ir2 0.00001).

### Processing and annotation of 10x Genomics ${5}^{{\prime} }$ gene expression and CITE-seq data

Data processing was performed in Seurat v5.2.1^46^. A 10x Genomics feature barcode HDF5 matrix with Gene Expression and antibody capture (ADT) counts was imported; a Seurat object was created from RNA counts (min.cells = 3, min.features = 200), and ADT was added as a separate assay. Low-quality cells were removed by requiring 200-4000 detected genes and < 10 mitochondrial RNA. RNA data were log-normalized (NormalizeData), variable features identified (FindVariableFeatures), scaled (ScaleData), and reduced with PCA. A shared nearest-neighbor graph was built on the first 10 PCs, clusters were called with the Louvain algorithm at resolution 0.8, and UMAP was computed on the same 10 PCs. ADT data were normalized with centered log-ratio (CLR, margin = 2). Cell types were assigned by label transfer to a multimodal PBMC reference^46^ using Seurat’s SCT-based anchor mapping. Barcodes for selected clusters and predicted cell types were exported for downstream analyses.

### Reporting summary

Further information on research design is available in the Nature Portfolio Reporting Summary linked to this article.

## Supplementary information

Supplementary Information

Reporting Summary

Transparent Peer Review file

## Data Availability

The datasets used in this study are publicly available. Dataset I RNA-seq data are available in GEO under accession GSE116500. Dataset II and Dataset III are available in ArrayExpress under accessions E-MTAB-4825 and E-MTAB-11452, respectively. Dataset IV is available from the 10x Genomics datasets portal [10x Genomics portal]. Dataset V RNA-seq data are available in the SRA under BioProject PRJNA783770. Matched Sanger-derived sequences used for validation are provided in Supplementary Table 3 of Andreani et al. ^8^. The BCR template sequences with increasing numbers of nucleotide substitutions are provided in Supplementary Table 4 of the same study. Source Data are provided with this paper.

## Code availability

The code used in this study is freely available at https://github.com/neuroimmunology-uio/gather and archived at Zenodo 10.5281/zenodo.20206619^47^.
