# Supplementary material for: GATHeR: graph-based accurate tool for immunoglobulin heavy- and light-chain reconstruction
Source: Nat Commun. 2026 Jun 10;17:7371. doi: 10.1038/s41467-026-74272-w (PMC13402621; doi:10.1038/s41467-026-74272-w)
Supplement: Supplementary file 1 — Supplementary Information [file 41467_2026_74272_MOESM1_ESM.pdf]

Supplementary Information

GATHeR: Graph-based Accurate Tool for Immunoglobulin HEavy- and Light-chain Reconstruction

Seyedmojtaba Seyedraoufi[1], Mari Bergstøl Gornitzka[1], Andreas Lossius[1,2]

[1]Department of Molecular Medicine, Institute of Basic Medical Sciences, University of Oslo, Oslo, Norway
[2]Department of Neurology, Akershus University Hospital, Lørenskog, Norway

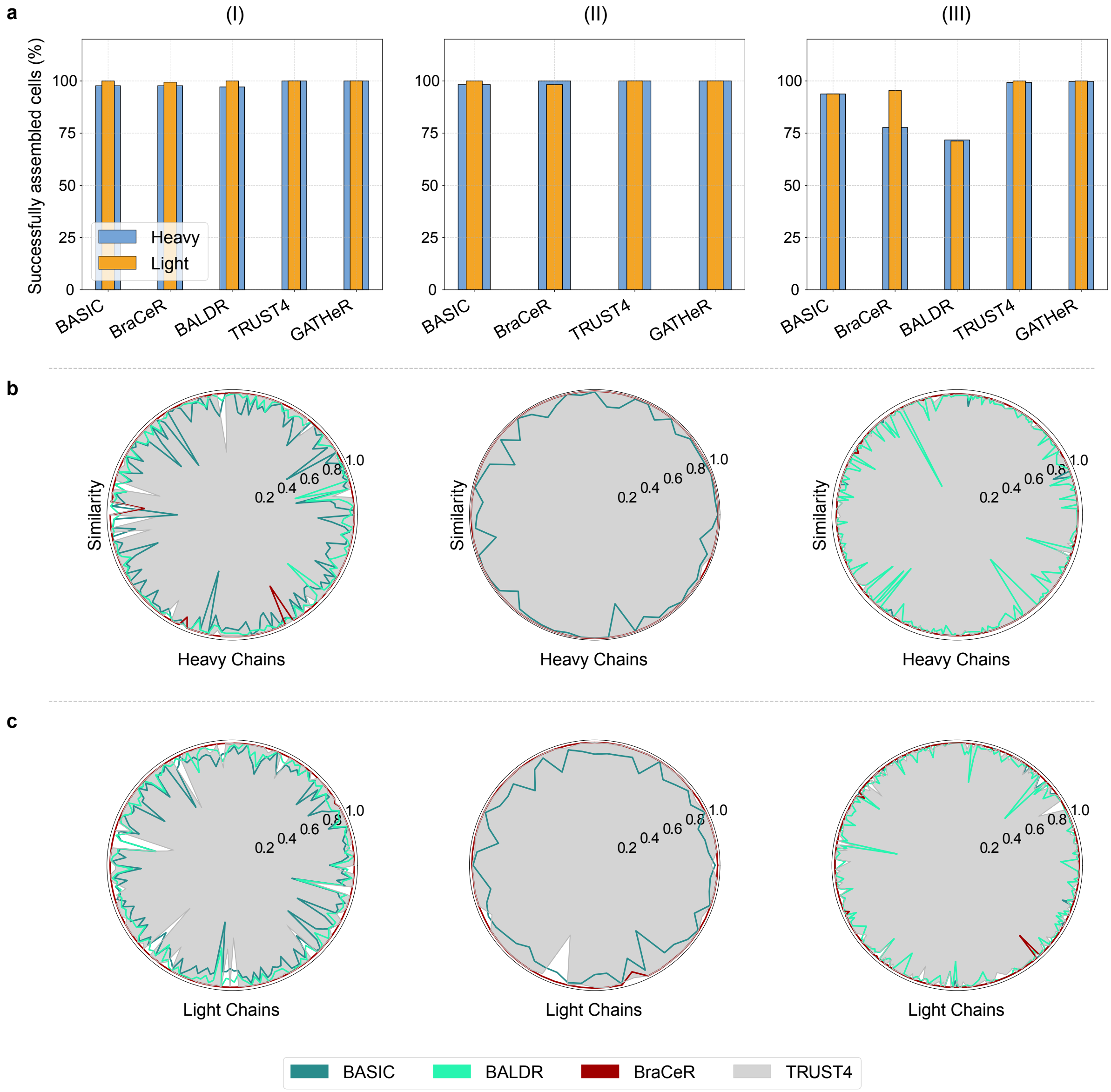

Supplementary Fig. 1. Performance benchmark of GATHeR against BASIC, BALDR, BraCeR and TRUST4. a, Bar plots showing, for each dataset (I–III, left to right), the fraction of cells in which each tool successfully reconstructed the heavy chain (IGH), the light chain (IGK/L) or both. **b,** Radar plots of heavy-chain sequence similarity and **c,** light-chain sequence similarity comparing contigs assembled by GATHeR with those from other tools across three datasets (I–III, left to right). Similarity is calculated as the number of identical nucleotides divided by the length of the shorter sequence, expressed as a fraction.

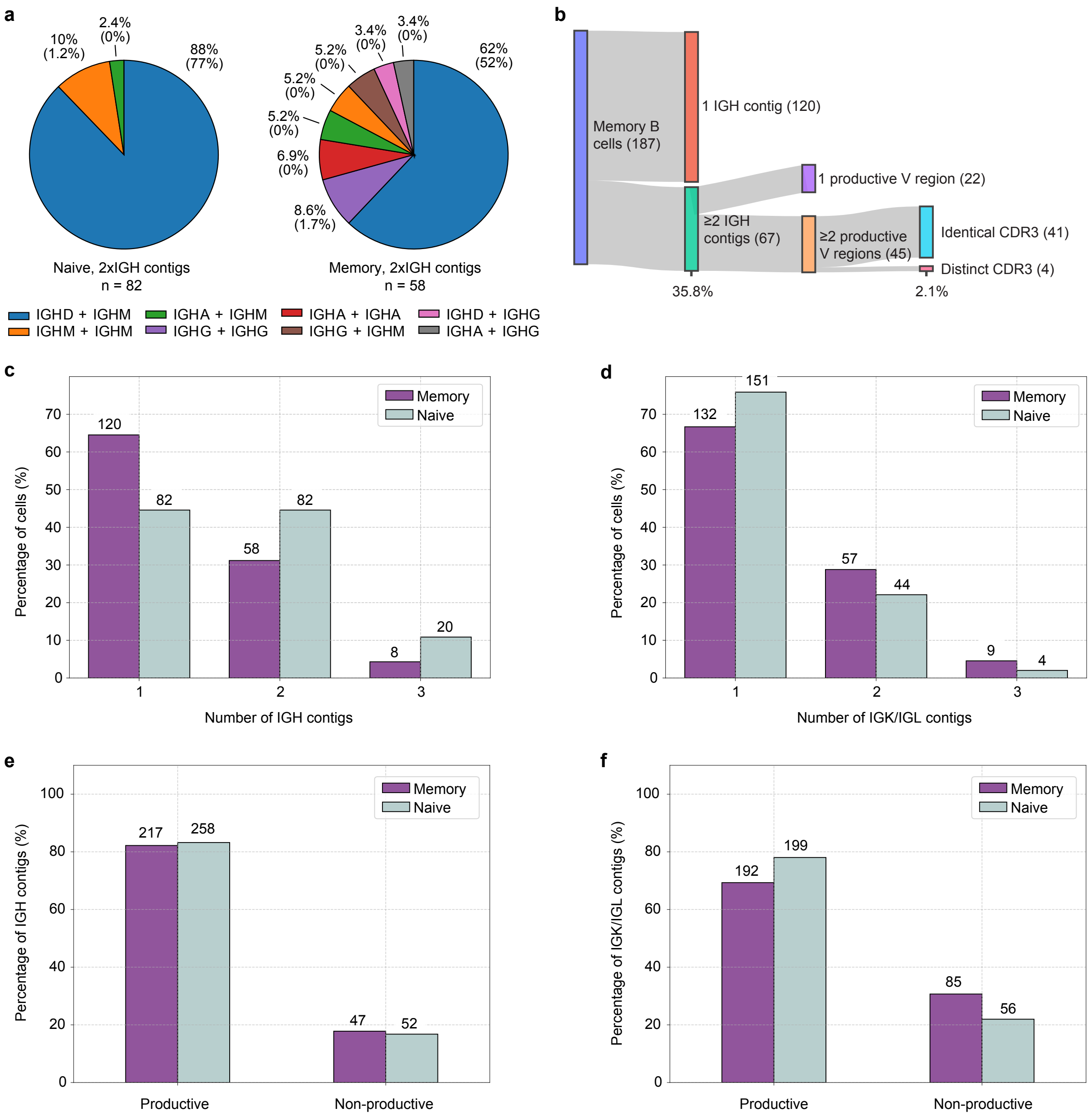

Supplementary Fig. 2. GATHeR identifies multiple heavy- and light-chain sequences per cell in a subset of naive and memory B cells in Dataset III. a, Isotype distribution among naive and memory B cells with two heavy-chain contigs; the proportion of cells in which the two heavy-chain contigs share an identical V(D)J junction (identical CDR3 nucleotide sequence) is shown in parentheses. **b,** Alluvial plot showing the distribution of IGH contig counts per cell, V region productivity (according to IgBLAST) and V region sharing (CDR3 identicality) in isotype-resolved memory B cells. **c,** Percentage of cells with 1, 2, or 3 reconstructed heavy-chain contigs and **d,** percentage of cells with 1, 2, or 3 reconstructed light-chain contigs, shown separately for isotype-resolved naive and memory B cells. **e,** Percentage of productive and non-productive heavy-chain assemblies and **f,** light-chain assemblies.

Intron retention IgM

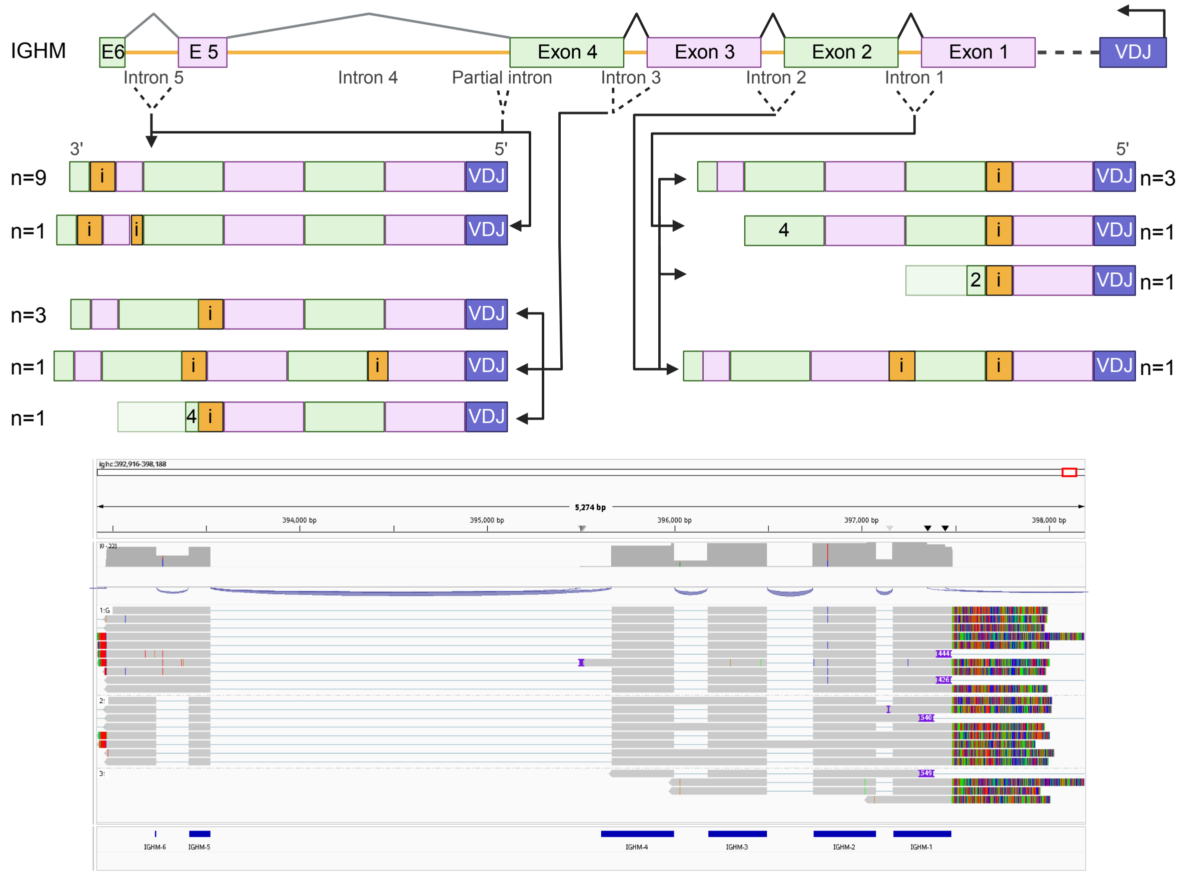

Intron retention IgD

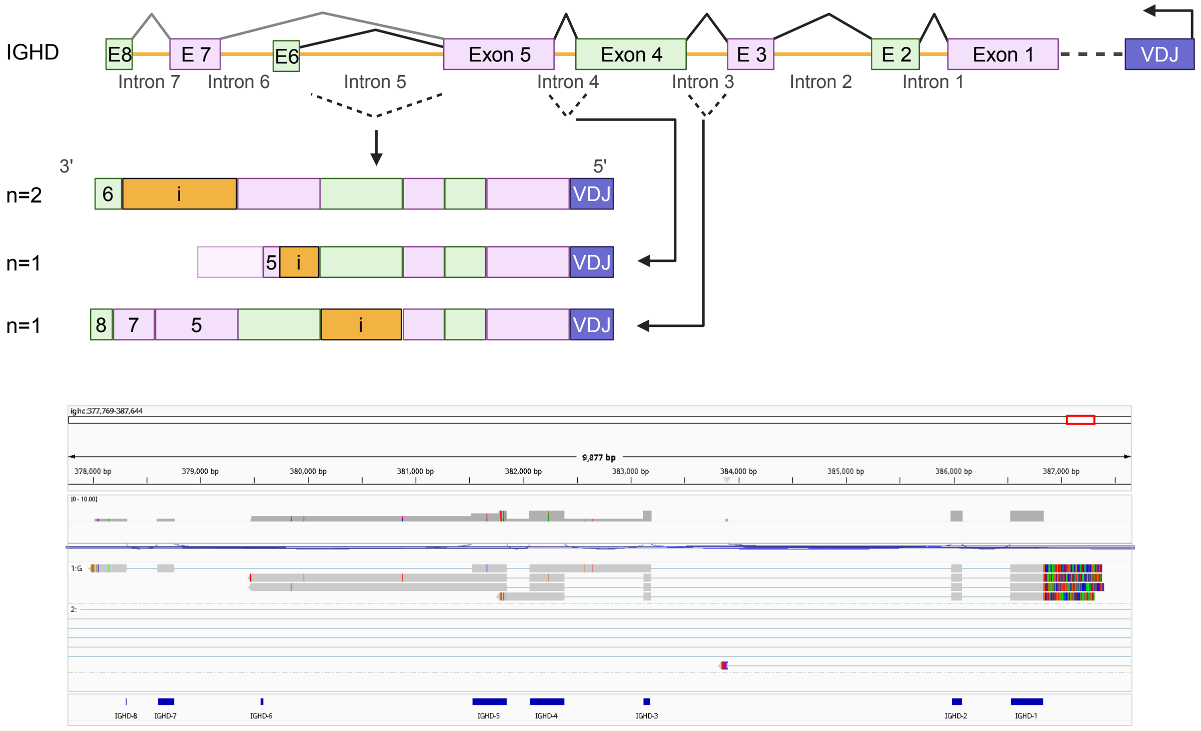

Supplementary Fig. 3 (continued on next page)

Intron retention IgG3

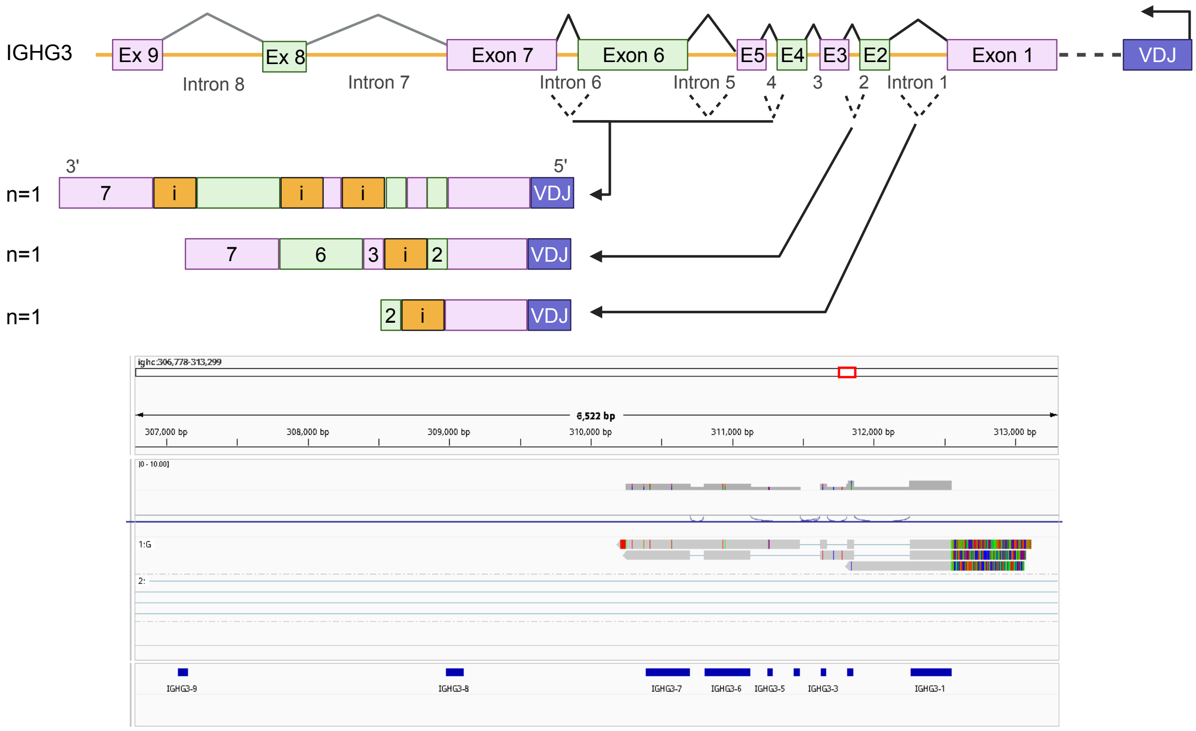

Intron retention IgG1

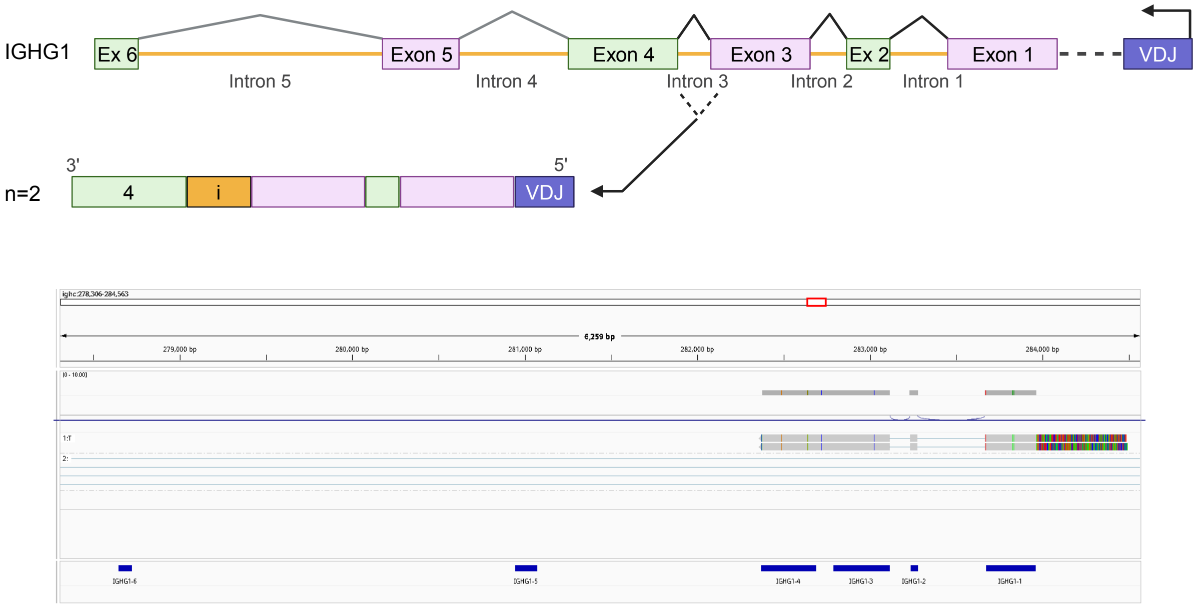

Supplementary Fig. 3 (continued on next page)

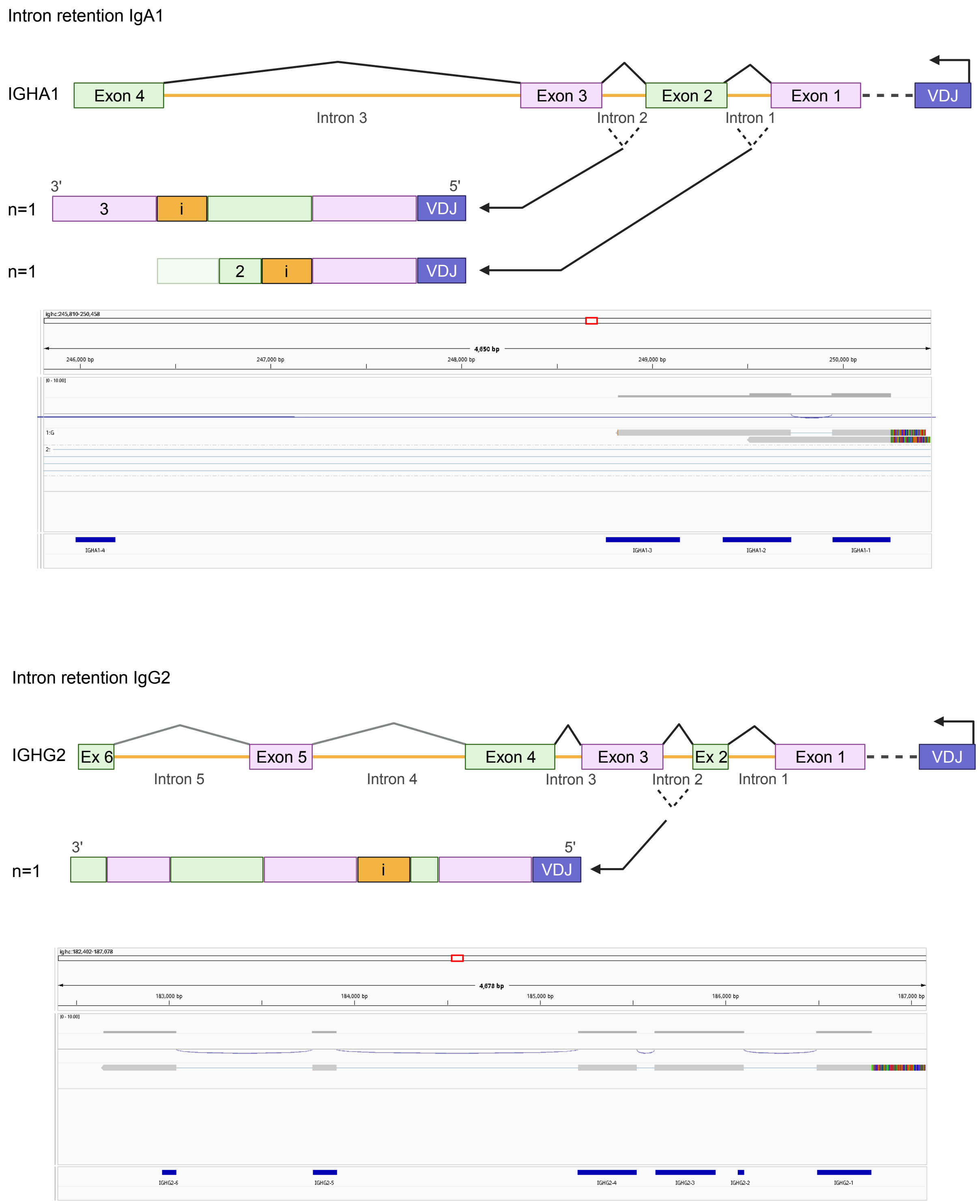

Supplementary Fig. 3. Schematic of candidate intron-retention events in GATHeR-assembled immunoglobulin heavy-chain transcripts from naive and memory B cells in Dataset III. For each isotype (IgM, IgD, IgG3, IgG1, IgA1, IgG2), all detected splice variants are shown; numbers to the left indicate how many reconstructed variants show that splicing pattern. Below, Integrative Genomics Viewer (IGV) snapshots show how the assembled transcripts align to the corresponding germline IGH gene. Created in BioRender. Lossius, A. (2026) https://BioRender.com/uw4xcrh.

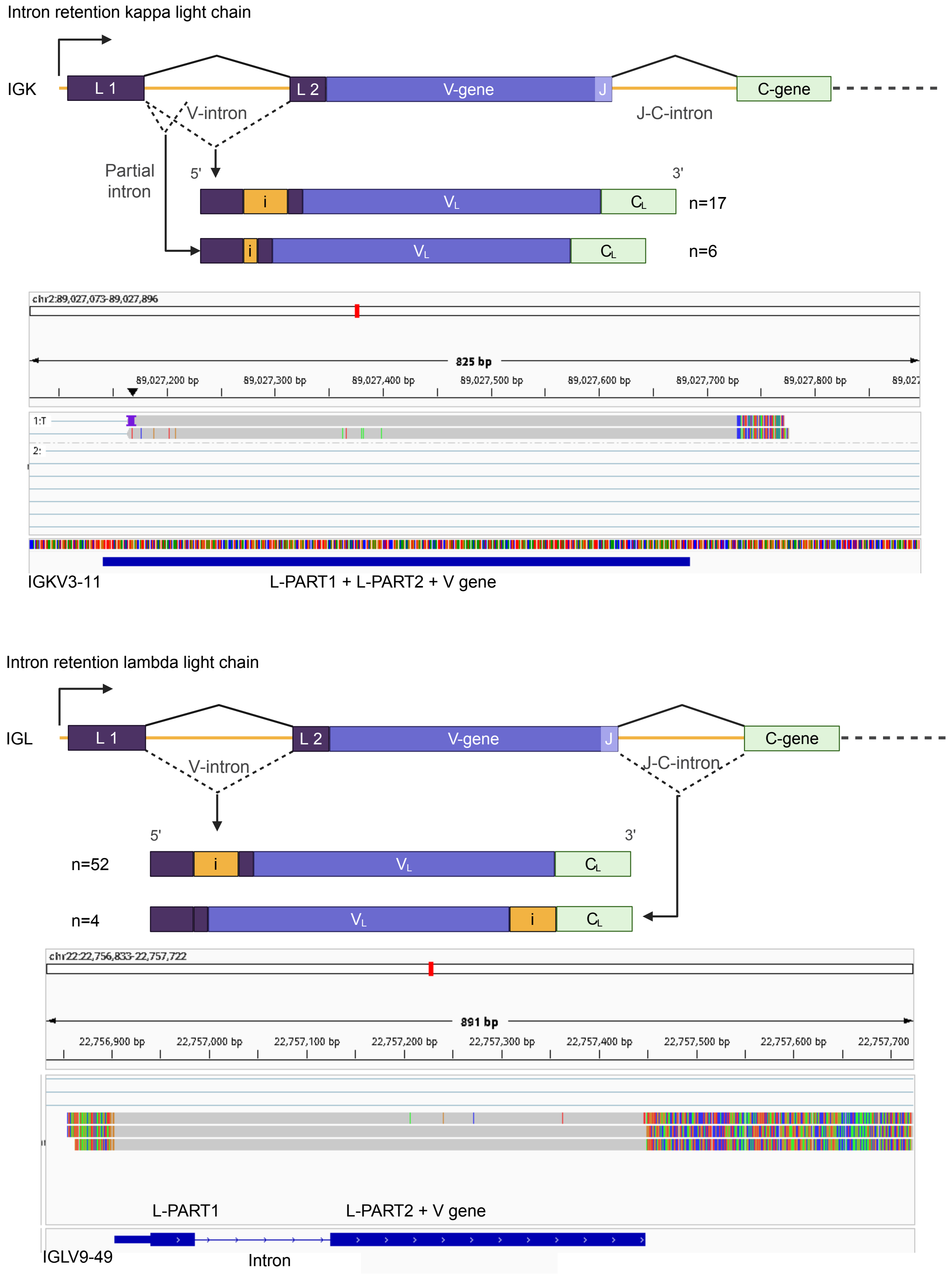

Supplementary Fig. 4. Schematic of candidate intron-retention events in GATHeR-assembled immunoglobulin light-chain transcripts from naive and memory B cells in Dataset III. κ chains exhibit partial and complete L–V (leader–variable) intron retention, whereas λ chains show L–V and J–C (joining–constant) intron retention. Numbers to the left indicate how many reconstructed variants show that splicing pattern. Integrative Genomics Viewer (IGV) snapshots below illustrate alignments of the assembled transcripts to the corresponding germline *IGK*/*IGL* genes. Created in BioRender. Lossius, A. (2026) https://BioRender.com/uw4xcrh.

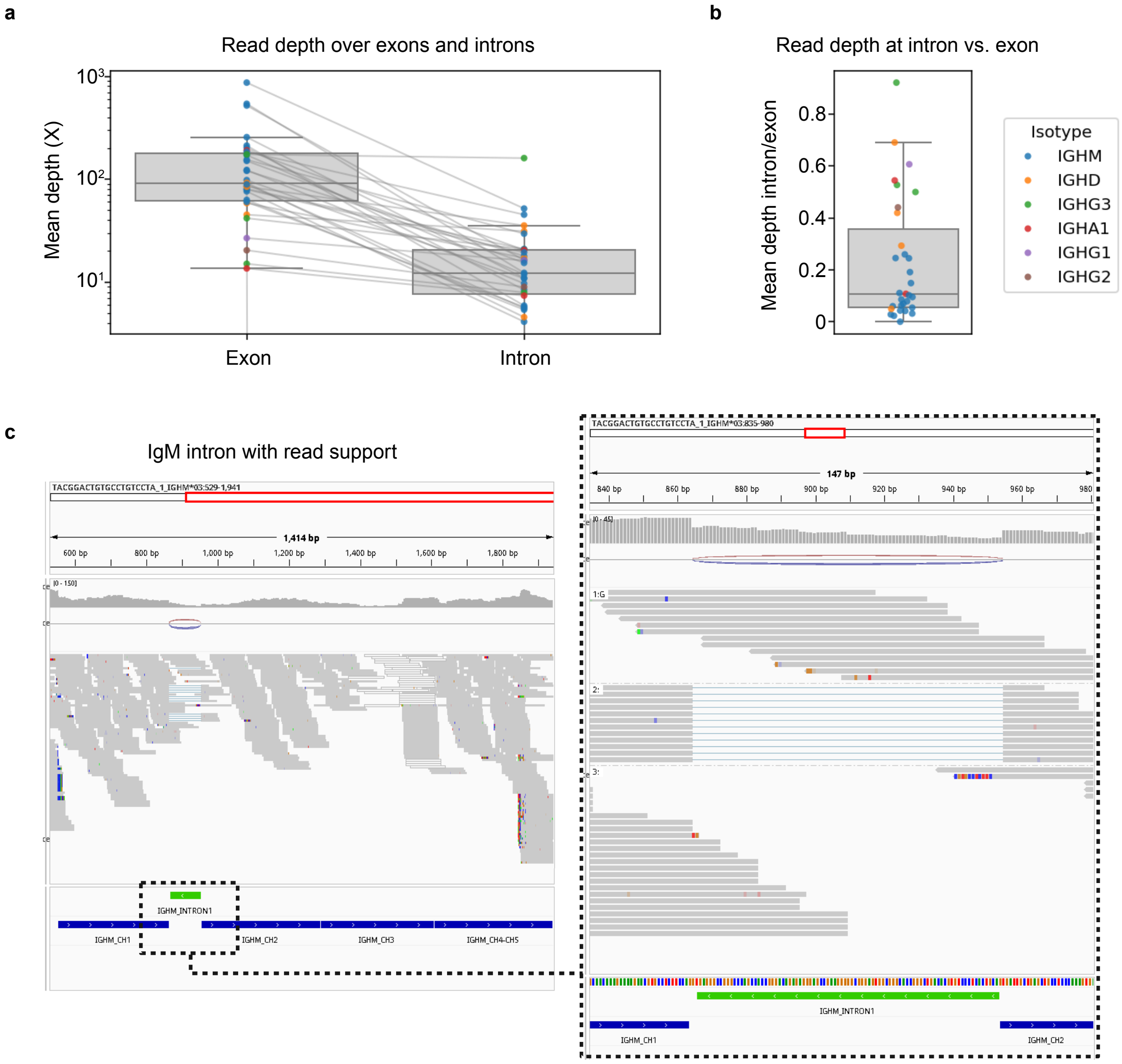

Supplementary Fig. 5. Sequencing reads support intronic seqeunces in GATHeR contigs. Reads from the cells with intron-containing contigs from Figure S3 were aligned back to their cognate contigs. IGHC exons and introns were annotated and used to define their borders before assessing the support for the intronic intervals. **a,** Dots represent mean read depth over exons or introns in individual intron-containing Ig heavy-chain contigs. Lines connect exon and intron depth from the same contig. **b,** Read depth at introns divided by mean exonic depth at the same contig gives measurement for relative support for intron-containing transcripts. **c,** IGV capture of read support for an IgM contig with intron between C_H1 and C_H2. Left image shows the entire IGHM region and right image is an enlargement of the intron sequence.

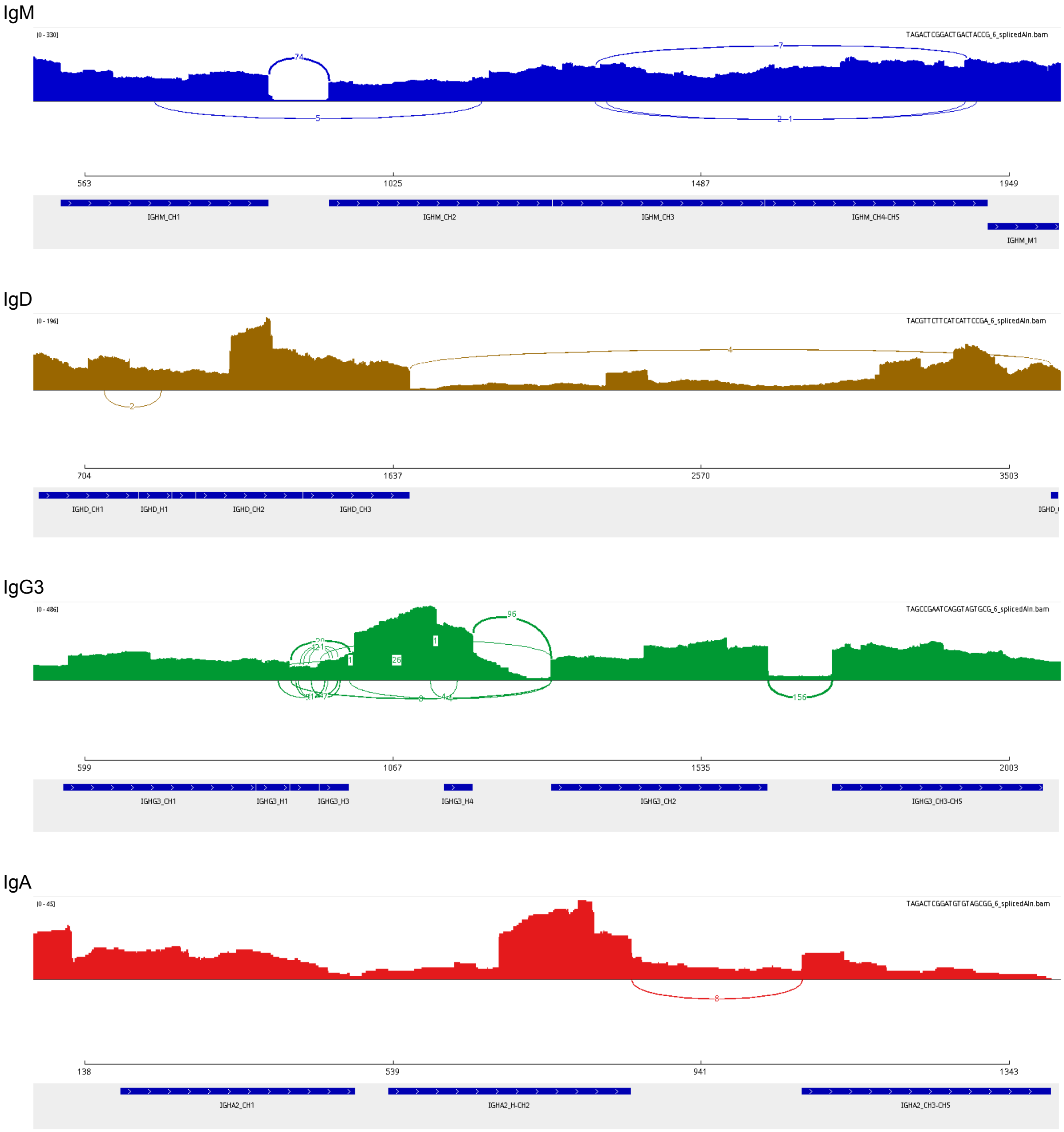

Supplementary Fig. 6. Sequencing reads support alternatively spliced constant regions in GATHeR Ig heavy-chain contigs. Reads from the cells with intron-containing contigs from Figure S3 were aligned back to their cognate contigs. IGHC exons and introns were annotated and used to define their borders. IGV was used to generate sashimi plots for contigs representative of each isotype.

donor	sequence_id	c_call	CH2_idn	CH4_idn	CH3_idn	CH1_idn	M1_idn
donor1	cell_1	IGHM*03-N	0.99	1.0	1.0	1.0	1.0
donor1	cell_2	IGHM*03-N	0.99	1.0	1.0	1.0	1.0
donor1	cell_3	IGHM*03-N	0.99	1.0	1.0	1.0	1.0
donor1	cell_4	IGHM*03	1.00	1.0	1.0	1.0	1.0
donor1	cell_5	IGHM*03-N	0.99	1.0	1.0	1.0	1.0
donor1	cell_6	IGHM*03	1.00	1.0	1.0	1.0	1.0
donor1	cell_7	IGHM*03-N	0.99	1.0	1.0	1.0	1.0
donor2	cell_1	IGHM*03	1.00	1.0	1.0	1.0	1.0
donor2	cell_2	IGHM*03	1.00	1.0	1.0	1.0	1.0
donor2	cell_3	IGHM*03	1.00	1.0	1.0	1.0	1.0
donor2	cell_4	IGHM*03	1.00	1.0	1.0	1.0	1.0
donor2	cell_5	IGHM*03	1.00	1.0	1.0	1.0	1.0
donor2	cell_6	IGHM*03	1.00	1.0	1.0	1.0	1.0
donor2	cell_7	IGHM*03	1.00	1.0	1.0	1.0	1.0

Supplementary Table 1. Simplified output table from GATHeR analysis of constant regions in assembled heavy chains from 14 naive B cells derived from two donors in Dataset III. The novel allele in donor 1 is annotated with the suffix "-N". Columns `CH2_idn`, `CH4_idn`, `CH3_idn`, `CH1_idn`, and `M1_idn` indicate the sequence identity of the assembled regions relative to the IMGT database.

Dataset	Platform	Species	Material	N	Source
V	Smart-seq2 + Sanger	Human	Memory B cells (matched RNA-seq and Sanger BCRs)	72	Andreani *et al.* (2022), doi:10.1093/nargab/lqac049
VI	In silico Illumina	Human	BCR templates (100 each IGH/IGK/IGL) with simulated SHM (15/30/45/60 substitutions per sequence)	1200	This study; based on Andreani *et al.* (2022) doi:10.1093/nargab/lqac049

Supplementary Table 2. Additional datasets used for orthogonal validation and SHM robustness analyses. Summary of the independent Smart-seq2 memory B-cell dataset with matched Sanger-derived BCR sequences and the in silico Illumina SHM stress-test dataset used in Figure 3B–C.

barcode	10x IGH (P/NP)	GATHeR IGH (P/NP)	10x NP length (bp)	GATHeR NP length (bp)	NP identity (%)
AGGGAGTAGGCTAGGT	2/1	2/1	454	332	100.0
CAGCTGGGTAGGAGTC	2/1	2/0	–	–	–
CCACTACCAGGGTATG	2/2	2/2	347,437	141,211	100.0,100.0
CCGTGGAAGGTGCAAC	2/1	2/1	581	114	99.1
CGAGCCACAGTAGAGC	2/1	2/0	553	–	–
CGATTGAAGCGATATA	2/1	2/1	639	244	100.0
CGGTTAAAGCCAGAAC	2/1	2/1	426	267	100.0
GGCCGATCAAGTTAAG	2/1	2/0	492	–	–
GGGTTGCGTCCTCCAT	2/1	1/1	431	160	100.0
GTGGGTCCAGACAGGT	2/1	2/0	451	–	–
TACAGTGTCGAACTGT	2/1	2/1	308	150	100.0
TACTTACGTCTAACGT	2/1	2/0	429	–	–
TGGGAAGTCACCGGGT	2/1	2/1	459	327	100.0

Supplementary Table 3. Cross-check of additional non-productive IGH calls using the matched 10x V(D)J reference. For each barcode, we report the number of IGH calls (productive/non-productive; P/NP) in the 10x V(D)J reference and recovered by GATHeR from the matched 5′ gene-expression data. Non-productive (NP) contig lengths are shown for the 10x reference and for the corresponding NP contigs recovered by GATHeR; NP identity is reported as percent identical nucleotides over the shorter aligned length. Barcodes are shown without the trailing "-1" suffix for readability.
