# Supplementary material for: GATHeR: graph-based accurate tool for immunoglobulin heavy- and light-chain reconstruction
Source: Nat Commun. 2026 Jun 10;17:7371. doi: 10.1038/s41467-026-74272-w (PMC13402621; doi:10.1038/s41467-026-74272-w)
Supplement: Supplementary file 3 — Transparent Peer Review file [file 41467_2026_74272_MOESM3_ESM.pdf]

nature portfolio

Peer Review File

GATHeR: Graph-based Accurate Tool for Immunoglobulin HEavy- and Light-chain Reconstruction

Corresponding Author: Dr Andreas Lossius

This file contains all reviewer reports in order by version, followed by all author rebuttals in order by version.

Version 0:

Reviewer comments:

Reviewer #1

(Remarks to the Author)
The authors present GATHeR, a new tool for reconstructing full-length B-cell receptor sequences from scRNA-seq data. However, the manuscript faces several critical challenges as a methods paper, the most central being insufficient novelty.
(1) The work represents a well-executed engineering effort but lacks a fundamental conceptual or algorithmic innovation. Its improvements over tools like TRUST4 seem more a consequence of a divergent design goal and parameter optimization rather than a technological leap. This does not meet the bar for originality expected for a high-impact methods paper.
(2) The benchmarking focuses on assembly success rate and sequence similarity but lacks a deeper evaluation of assembly accuracy. There is no report or discussion of chimeric sequence rates, misassembly rates, or INDEL errors.
(3) The benchmark datasets used are relatively "clean". There is a lack of testing in more challenging scenarios, such as Cancer samples, where extremely high somatic hypermutation rates might disrupt k-mer-based assembly.
(4) The current tool only supports BCRs and not T-cell receptors, limiting its appeal for comprehensive immune repertoire analysis.

(Remarks on code availability)

Reviewer #2

(Remarks to the Author)
A manuscript “GATHeR: Graph-based Accurate Tool for Immunoglobulin HEavy- and Light-chain Reconstruction” presents a development of a novel open-source computational pipeline for full-length B-cell receptor (BCR)/immunoglobulin sequence assembly from single-cell RNA-seq data.

The work addresses a critical limitation in current BCR reconstruction methods, namely, incomplete constant-region reconstruction, which prevents accurate isotype, subclass and allele identification. It employs compacted de Bruijn graphs for de novo assembly, elegantly integrating rnaSPAdes for handling cases (especially naïve/memory B cells) in samples with uneven or low sequence coverage. Importantly, GATHeR allows membrane-bound vs secreted immunoglobulin isoform discrimination which is a limitation is other BCR assembly tools as well as has post-processing modules for clonality analyses and phylogenetic tree reconstruction making it an attractive platform to perform immunoglobulin assembly all the way from raw reads (supporting data from both most common platforms Smart-seq2/3 and 10x) to clonotype analyses. Moreover, it basically eliminates the need for V(D)J enrichment in 10x experiments thus saving samples, time and costs and enabling retrospective analyses of existing scRNA-seq datasets. The authors used four datasets to benchmark GATHeR against other state-of-art methodologies, showing its superiority in retained intron and multiple chain per cell detection which could make GATHeR a preferred tool for BCR/Immunoglobulin assembly tasks.

However, although the work presents an important advancement in BCR repertoire analyses, the authors need to make major improvements especially in the validation of the novel findings before publishing.

Major comments

Comment 1: The authors state that they selected tools to benchmark GATHeR against “based on favorable performance in a recent comparative study” referencing Andreani et al. 2022 manuscript. The referenced manuscript assessed more tools, including BALDR which was found to be one of the top performers in the above-mentioned study but was omitted in the current study. Authors should include results from BALDR in the manuscript to support their claims of GATHeR being “a robust, end-to-end solution” for BCR assembly.

Comment 2: The authors claim that GATHeR can identify intron-retention events, providing “the first evidence for intron retention within heavy-chain transcripts” at the same time mentioning that “we cannot exclude some contribution from unspliced nuclear pre-mRNA”. To make these claims, they should provide an orthogonal validation (qPCR, Sanger) to prove that these are true events and not just artefacts of sequence assembly algorithm used by GATHeR to support that, especially, knowing that other tools, and especially TRUST4 failed to identify them.

Comment 3: The authors present data showing that GATHeR identifies multiple chains in one cell at the same time saying that observation of multiple chains in “some instances may reflect technical artifacts”. Again, they should provide orthogonal validation to show that e.g. non-productive chains are true and not just an artefact in order to make these claims.

Comment 4: The authors should present the chain reconstruction accuracy on gene and sequence level of GATHeR in comparison to Sanger sequencing data, e.g. using the data available in the Canazar et al. 2017 dataset (reference 8) or other dataset to show how their tool compares with the ground truth. At the moment it is not clear if, for example, GATHeR reconstructs longer constant domains at the expense of the lower accuracy in variable domain reconstruction.

Minor comments
Comment 1: Figure names should be the same throughout the manuscript and the supplement, e.g. Supplementary Figure 1, Supplementary Figure 2A, etc. OR Figure S1, Figure S2A.

Comment 2: How sensitive is GATHeR to k-mer size?

Comment 3: $\phi(P) = \Sigma w(u)/max(|P|$, why do authors normalize by the longest path rather than the current path length? This could bias toward longer paths with lower average weight.

Comment 4: The authors say that to create Dataset III, they randomly sampled 400 cells from the published dataset. They should provide a file with cell/sample/file IDs that were used in the benchmark.

Comment 5: Manuscript lacks information on how secondary contigs are ranked and reported by GATHeR.

(Remarks on code availability)

Version 1:

Reviewer comments:

Reviewer #1

(Remarks to the Author)
None

(Remarks on code availability)
None

Reviewer #2

(Remarks to the Author)
I would like to thank the authors for their efforts in revising the manuscript and for incorporating the requested additional analyses and clarifications. The inclusion of the two new validation datasets using Sanger and VDJ sequencing and the deeper characterization of intron retention events significantly strengthens the technical claims of the paper.

1. I have a major concern regarding the results described in lines 180-186, regarding the double heavy chain assembly in memory B cells. Biologically, it is almost impossible to have 2 productive heavy chains in a memory B cell under normal conditions as those cells would not pass the developmental checkpoints and would die. Previous studies reported <1% of the cells with two productive heavy chains whereas the current work states that "two or more IGH contigs were detected in 35% of annotated memory B cells (Supplementary Fig. 2c), and two or more light-chain contigs in 24% (Supplementary Fig. 2d). Among additional contigs, 17% of heavy- <...>-chain assemblies were classified as non-productive" which is fewer than a half of all double heavy chains.

Authors should investigate and explain why they find such a substantial amount of memory B-cells that have two productive heavy chains that is neither backed by the previous literature nor the current knowledge about the B-cell Biology. They should also put the absolute cell numbers in the Supplementary figures 2a-f.

They should also provide double heavy & light chain numbers including productive/non-productive chains and figures similar to Supplementary figure 2 for other datasets mentioned in the study and discuss how these compare with previous studies/other BCR assembly tools.

2.Regarding the datasets presented in Table 1, there were more cells in the original datasets and and publications that investigated them than the cell numbers shown in Table 1. Why were some cells excluded and what was the rationale behind the filtering approach that was employed? This has to be stated in the manuscript.

3. By looking at the new Figure 3, it is clear that GATHER shows very similar performance to other published tools regarding constant-region assembly. This should be mentioned in the discussion.

(Remarks on code availability)

Version 2:

Reviewer comments:

Reviewer #2

(Remarks to the Author)
Dear Authors,

Thank you very much for your responses and extensive revisions to the manuscript that have resolved my main concerns and significantly strengthened the work.

I appreciate the effort put into these revisions. I have no further suggestions and will recommend publication of this article to the editor.

All the best with your future work!

(Remarks on code availability)

Open Access This Peer Review File is licensed under a Creative Commons Attribution 4.0 International License, which permits use, sharing, adaptation, distribution and reproduction in any medium or format, as long as you give appropriate credit to the original author(s) and the source, provide a link to the Creative Commons license, and indicate if changes were made.
In cases where reviewers are anonymous, credit should be given to 'Anonymous Referee' and the source.
The images or other third party material in this Peer Review File are included in the article's Creative Commons license, unless indicated otherwise in a credit line to the material. If material is not included in the article's Creative Commons license and your intended use is not permitted by statutory regulation or exceeds the permitted use, you will need to obtain

permission directly from the copyright holder.
To view a copy of this license, visit https://creativecommons.org/licenses/by/4.0/

Reviewer #1

The authors present GATHeR, a new tool for reconstructing full-length B-cell receptor sequences from scRNA-seq data. However, the manuscript faces several critical challenges as a methods paper, the most central being insufficient novelty.
(1) The work represents a well-executed engineering effort but lacks a fundamental conceptual or algorithmic innovation. Its improvements over tools like TRUST4 seem more a consequence of a divergent design goal and parameter optimization rather than a technological leap. This does not meet the bar for originality expected for a high-impact methods paper.

Response 1.1: Thank you for the opportunity to clarify the conceptual and algorithmic novelties of GATHeR. In the revised manuscript, we make explicit that GATHeR is built around a distinct reconstruction endpoint compared with V(D)J/CDR3-centric methods: isoform-aware, full-length immunoglobulin transcripts with extended constant-region coverage, including transmembrane/cytoplasmic-tail exons. This enables membrane-versus-secreted calling, subclass/allele/isoform annotation, and identification of splice variants directly from standard scRNA-seq data.

Methodologically, this is not a parameter-tuned variant of existing tools. GATHeR assembles raw scRNA-seq reads into a compacted de Bruijn graph and uses a dedicated contig-recovery strategy: it decomposes the graph into weakly connected components and traverses each component using support-guided path scoring that prioritizes long, well-supported paths, thereby favoring contiguous full-length reconstruction (including constant-region sequence) when evidence exists. Candidate heavy- and light-chain contigs are then identified and annotated using IMGT exon coordinates as a detector/annotator rather than as a strict assembly template, reducing dependence on a fixed reference transcript structure and improving robustness to sequence divergence (including elevated SHM burdens).

By contrast, V(D)J/CDR3-first tools such as TRUST4 typically start from V/J-anchored read selection and extension, reflecting a different reconstruction endpoint and therefore a different algorithmic strategy. Accordingly, the contrast is clearest when the goal includes full-length/constant-region sequence and isoform-level information, or when divergence from germline is high. Consistent with the latter, our controlled SHM stress test shows clear performance differences at higher mutation burdens (new Figure 3C; see also Response 1.3).

We have revised the Introduction and Methods to highlight the novelty of the design choices and their practical implications (Introduction, lines 44–53; Methods, lines 312–317).

> *(2) The benchmarking focuses on assembly success rate and sequence similarity but lacks a deeper evaluation of assembly accuracy. There is no report or discussion of chimeric sequence rates, misassembly rates, or INDEL errors.*

Response 1.2: We agree and have expanded the benchmarking to include a deeper, ground-truth–based evaluation of assembly accuracy and error modes. Specifically, we performed orthogonal validation against matched Sanger-derived BCR sequences in two independent plate-based datasets (Dataset I and an additional Smart-seq2 memory B-cell dataset with Sanger ground truth; Supplementary Table S1). We now report (i) gene-level agreement (V/D/J and, where applicable, C-region/isotype), (ii) nucleotide-level accuracy (local identity and truth coverage), and (iii) explicit error signatures, including INDEL rates (indels per kb). To capture misassembly/chimeric failure modes, we additionally report the fraction of reconstructions that are discordant with the matched Sanger ground truth in their V–D–J composition ("VDJ-set discordance"), which provides a conservative proxy for chimeric or misassembled contigs. These results are presented in the new Figure 3A–B and described in the new Results section "GATHeR matches Sanger-derived ground truth and remains accurate under increasing somatic hypermutation (SHM)" (lines 122–152).

(3) The benchmark datasets used are relatively "clean". There is a lack of testing in more challenging scenarios, such as Cancer samples, where extremely high somatic hypermutation rates might disrupt k-mer-based assembly.

Response 1.3: To address the concern that our benchmarks are relatively “clean”, we added an explicit somatic hypermutation (SHM) stress test with known ground truth. This stress test directly addresses the reviewer’s concern about robustness under high sequence divergence, where reduced exact k-mer sharing between reads can challenge reconstruction. Following the strategy of Andreani et al. (NAR Genomics & Bioinformatics, 2022), we constructed an in silico Illumina dataset by simulating scRNA-seq reads from heavy- and light-chain template sequences with graded SHM burdens of 15, 30, 45, and 60 substitutions per sequence (Methods, lines 394–408). We then ran GATHeR and comparator methods on identical inputs. As shown in the new Figure 3C, GATHeR remains robust across increasing SHM, whereas some comparator methods show reduced success and/or declining sequence similarity at higher mutation burdens. These SHM stress-test results are described in the new Result section (lines 154–161) and discussed in the context of practical failure modes at high SHM (Discussion, lines 296–299).

(4) The current tool only supports BCRs and not T-cell receptors, limiting its appeal for comprehensive immune repertoire analysis.

Response 1.4: We agree that adding TCR reconstruction would broaden the utility of the software. However, the present study is intentionally scoped to BCRs, where constant-region–resolved, isoform-aware full-length reconstruction from standard scRNA-seq remains an under-served problem. Accordingly, GATHeR is designed and benchmarked for paired, full-length heavy/light assemblies with extended constant-region coverage enabling isotype/subclass assignment and membrane-versus-secreted discrimination. We now explicitly acknowledge the lack of TCR support as a limitation in the Discussion (lines 299–303) and note that extending the framework to TCR α/β would require locus-specific adaptation and dedicated benchmarking, which is beyond the scope of this manuscript.

Reviewer #2

A manuscript "GATHeR: Graph-based Accurate Tool for Immunoglobulin HEavy- and Light-chain Reconstruction" presents a development of a novel open-source computational pipeline for full-length B-cell receptor (BCR)/immunoglobulin sequence assembly from single-cell RNA-seq data.

The work addresses a critical limitation in current BCR reconstruction methods, namely, incomplete constant-region reconstruction, which prevents accurate isotype, subclass and allele identification. It employs compacted de Bruijn graphs for de novo assembly, elegantly integrating rnaSPAdes for handling cases (especially naïve/memory B cells) in samples with uneven or low sequence coverage. Importantly, GATHeR allows membrane-bound vs secreted immunoglobulin isoform discrimination which is a limitation is other BCR assembly tools as well as has post-processing modules for clonality analyses and phylogenetic tree reconstruction making it an attractive platform to perform immunoglobulin assembly all the way from raw reads (supporting data from both most common platforms Smart-seq2/3 and 10x) to clonotype analyses. Moreover, it basically eliminates the need for V(D)J enrichment in 10x experiments thus saving samples, time and costs and enabling retrospective analyses of existing scRNA-seq datasets. The authors used four datasets to benchmark GATHeR against other state-of-art methodologies, showing its superiority in retained intron and multiple chain per cell detection which could make GATHeR a preferred tool for BCR/Immunoglobulin assembly tasks.

However, although the work presents an important advancement in BCR repertoire analyses, the authors need to make major improvements especially in the validation of the novel findings before publishing.

Major comments

Comment 1: The authors state that they selected tools to benchmark GATHeR against “based on favorable performance in a recent comparative study” referencing Andreani et al. 2022 manuscript. The referenced manuscript assessed more tools, including BALDR which was found to be one of the top performers in the above-mentioned study but was omitted in the current study. Authors should include results from BALDR in the manuscript to support their claims of GATHeR being “a robust, end-to-end solution” for BCR assembly.

Response 2.1: We thank the reviewer for this suggestion. BALDR was highlighted as a strong performer in Andreani et al. (2022), and we agree that including it strengthens the benchmark. In the revised manuscript, we have added BALDR to our benchmarking and report its performance on all datasets for which it is applicable (new Figures 2–4; Supplementary Figure 1). BALDR is designed for reconstructing human (and rhesus macaque) immunoglobulin/BCR sequences and is not designed or validated for mouse data; we therefore did not evaluate BALDR on Dataset II (mouse). We have clarified these applicability constraints in the revised Results and in the relevant figure legends. Overall, inclusion of BALDR on the applicable datasets is consistent with our conclusions regarding GATHeR’s performance.

Comment 2: The authors claim that GATHeR can identify intron-retention events, providing “the first evidence for intron retention within heavy-chain transcripts” at the same time mentioning that “we cannot exclude some contribution from unspliced nuclear pre-mRNA”. To make these claims, they should provide an orthogonal validation (qPCR, Sanger) to prove that these are true events and not just artefacts of sequence assembly algorithm used by GATHeR to support that, especially, knowing that other tools, and especially TRUST4 failed to identify them.

Response 2.2: We agree that orthogonal validation (qPCR, Sanger sequencing) would be the most definitive approach. However, we do not have access to the original biological material from the relevant samples/cells required to perform these experiments. We have therefore revised the manuscript to avoid overstatement: we now refer to these as candidate intron-containing heavy-chain transcripts with read-level support and have removed/toned down language implying definitive intron retention (Abstract,

line 19; Results, lines 188–208; Discussion, lines 237 and 255–269). We also explicitly note that we cannot exclude contributions from unspliced nuclear pre-mRNA (Discussion, lines 262–264).

To address the reviewer's concern that these signals could reflect assembly artefacts, we added assembly-independent read-level support from the corresponding cells. Briefly, R1/R2 reads were aligned back to the GATHeR contigs together with a transcriptome decoy reference to reduce spurious alignments. We then annotated constant-region introns/exons on the contigs and quantified read depth across these intervals (new Supplementary Figure 5A–B). As expected, exonic read depth exceeded intronic depth, but we nevertheless observed consistent coverage across the intron body. Importantly, we also detected multiple reads spanning the exon–intron and intron-exon boundaries, and we provide an IgM example in the new Supplementary Figure 5C. In addition, sashimi plots across multiple isotypes show junction-spanning support consistent with intron-containing transcripts (new Supplementary Figure 6). Together, these analyses indicate that the reported intron-containing reconstructions are not introduced solely by contig assembly.

> *Comment 3: The authors present data showing that GATHeR identifies multiple chains in one cell at the same time saying that observation of multiple chains in "some instances may reflect technical artifacts". Again, they should provide orthogonal validation to show that e.g. non-productive chains are true and not just an artefact in order to make these claims.*

Response 2.3: We agree that reconstruction of multiple chains per cell can reflect true biology, barcode-level technical artifacts in scRNA-seq (e.g., multiplets or ambient RNA contamination), or assembly artefacts. We have revised the manuscript to explicitly separate these cases and to avoid over-interpreting multi-chain calls.

To address the reviewer's request for independent validation, we utilized the targeted 10x V(D)J–enriched library generated with dedicated primers (separate from the 5′ GEX library used for assembly) and the corresponding V(D)J annotations provided by 10x Genomics for this dataset. Because these V(D)J calls

are obtained with an independent library preparation designed specifically for V(D)J detection, concordance between GATHeR assemblies from 5′ GEX and the 10x V(D)J annotations supports that the additional (including non-productive) rearrangements are present in the sample and are unlikely to be introduced by GATHeR's assembly alone.

Specifically, we analyzed a subset of cells in which the 10x Genomics V(D)J annotations reported at least two IGH calls, including one non-productive. In this subset, GATHeR detected all productive chains and 9/13 of the additional non-productive chains in the matched 5′ GEX data (Results, lines 223–225; new Supplementary Table 3). Together, these results support that the observed non-productive rearrangements are not solely assembly artifacts of GATHeR.

In naïve B cells, the dominant multi-IGH pattern identified by GATHeR corresponds to paired IGHM and IGHD constant-region isoforms sharing an identical VDJ junction (identical CDR3 nucleotide sequence), consistent with known IgM/IgD co-expression from a single rearranged VDJ exon. We now refer to these events as constant-region isoform co-expression, rather than independent multiple receptors (new Supplementary Figure 2A–B; Results, lines 172–176).

We also note that detection of an additional non-productive IgH transcript in the same cell is compatible with expected B-cell biology, as sequential rearrangements across the two alleles can yield a productive and a non-productive allele in the same cell. We therefore interpret non-productive contigs as rearrangement history rather than evidence of co-expression of multiple functional receptors (Results, lines 185–186; Discussion, lines 277–280). Conversely, barcodes containing multiple productive contigs with discordant V(D)J junctions can arise from technical factors (e.g., multiplets or ambient RNA) and are not used to support biological claims (Discussion, lines 280–283).

> *Comment 4: The authors should present the chain reconstruction accuracy on gene and sequence level of GATHeR in comparison to Sanger sequencing data, e.g. using the data available in the Canazar et al. 2017 dataset (reference 8) or other dataset to show how their tool*

compares with the ground truth. At the moment it is not clear if, for example, GATHeR reconstructs longer constant domains at the expense of the lower accuracy in variable domain reconstruction.

Response 2.4: We thank the reviewer for this important suggestion and agree that chain reconstruction accuracy should be evaluated against nucleotide-level ground truth. We therefore added orthogonal validation using matched Sanger-derived BCR sequences. Specifically, our Dataset I corresponds to the Canzar et al. 2017 Sanger-ground-truth dataset referenced by the reviewer. Notably, in this dataset a subset of Sanger sequences were generated from cloned vectors where the constant region is fixed in the vector so the cell-specific insert does not include the constant region, which excludes the constant-region segments from the independent ground-truth validation. We therefore additionally included an independent plate-based Smart-seq2 memory B-cell dataset with matched Sanger sequences (new Supplementary Table S1) to enable constant-region/isotype validation in a second cohort and experimental setting.

Across these data, we now report accuracy at both the gene and sequence level, including V/D/J gene agreement and nucleotide-level accuracy metrics against Sanger ground truth (local identity, truth coverage, and INDEL rates reported as indels per kb from V-segment alignments). For the new dataset, we additionally report constant-region/isotype agreement. Together, these analyses address the reviewer's concern about reconstruction accuracy and do not suggest that improved constant-region recovery is achieved at the expense of variable-region accuracy in the validated datasets. These results are presented in the new Figure 3A–B and described in the revised manuscript (Results, lines 122–152; Methods, lines 377–393).

Minor comments

Comment 1: Figure names should be the same throughout the manuscript and the supplement, e.g. Supplementary Figure 1, Supplementary Figure 2A, etc. OR Figure S1, Figure S2A.

Response 2.5: We have standardized figure naming and cross-referencing throughout the main manuscript and the Supplementary Information to follow Nature Communications style. We now use "Supplementary Fig. X" consistently (with panels a–c where relevant) and have removed mixed usage such as "Figure/Fig. S…".

Comment 2: How sensitive is GATHeR to k-mer size?

Response 2.6: We assessed sensitivity to k-mer size by varying the de Bruijn graph k while keeping all other parameters fixed. Performance was stable across a moderate range (k = 25–31), motivating k = 25 as a conservative default. Smaller k increased erroneous assemblies (particularly near transcript ends), whereas very large k increased fragmentation and shortened contigs under uneven or low coverage.

Comment 3: $\phi(P) = \Sigma w(u)/max(|P|$, why do authors normalize by the longest path rather than the current path length? This could bias toward longer paths with lower average weight.

Response 2.7: Thank you for this helpful point. We did not intend $\phi(P)$ to represent an "average weight per unitig". Rather, $\phi(P)$ is a component-normalized accumulated-support score. Because $max(|P|)$ is constant within each weakly connected component G', it does not affect the ranking of paths within that component; it is included only as a scaling factor. Using $\Sigma w(u)/|P|$ would convert the score into a true average and can strongly favor short, high-coverage fragments (often V(D)J/CDR3-only) over longer reconstructions extending into the constant region where coverage is naturally lower, which conflicts with our full-length objective. We have clarified this in the revised Methods ("Traversing and finding optimum paths"; lines 345–349).

Comment 4: The authors say that to create Dataset III, they randomly sampled 400 cells from the published dataset. They should provide a file with cell/sample/file IDs that were used in the benchmark.

Response 2.8: We agree. For full reproducibility, we now provide the complete set of the 400 selected Dataset III cell/barcode IDs used in the benchmark in the Source Data.

Comment 5: Manuscript lacks information on how secondary contigs are ranked and reported by GATHeR.

Response 2.9: We agree and have clarified this in the revised Methods, lines 362–365. Briefly, all contigs (including rnaSPAdes fallback contigs) are written to the FASTA output and a normalized weight reported in the FASTA header is used to rank and report primary and secondary contigs.

Reviewer #1

Reviewer #1 (Remarks to the Author):

None

Reviewer #1 (Remarks on code availability):

None

Response 1.1: We thank the reviewer for re-evaluating the revised manuscript and confirming that no further changes are required.

Reviewer #2

I would like to thank the authors for their efforts in revising the manuscript and for incorporating the requested additional analyses and clarifications. The inclusion of the two new validation datasets using Sanger and VDJ sequencing and the deeper characterization of intron retention events significantly strengthens the technical claims of the paper.

Response 2.0: We appreciate the reviewer's positive assessment and constructive feedback. We have revised the manuscript to address the remaining points raised below.

1. I have a major concern regarding the results described in lines 180-186, regarding the double heavy chain assembly in memory B cells. Biologically, it is almost impossible to have 2 productive heavy chains in a memory B cell under normal conditions as those cells would not pass the developmental checkpoints and would die. Previous studies reported <1% of the cells with two productive heavy chains whereas the current work states that "two or more IGH contigs were detected in 35% of annotated memory B cells (Supplementary Fig. 2c), and two or more light-chain contigs in 24% (Supplementary Fig. 2d). Among additional contigs, 17% of heavy-

<...>-chain assemblies were classified as non-productive" which is fewer than a half of all double heavy chains.

Authors should investigate and explain why they find such a substantial amount of memory B-cells that have two productive heavy chains that is neither backed by the previous literature nor the current knowledge about the B-cell Biology. They should also put the absolute cell numbers in the Supplementary figures 2a-f.
They should also provide double heavy & light chain numbers including productive/non-productive chains and figures similar to Supplementary figure 2 for other datasets mentioned in the study and discuss how these compare with previous studies/other BCR assembly tools.

Response 2.1: We agree that bona fide co-expression of two distinct productive heavy-chain rearrangements in memory B cells is expected to be rare. Our previous wording and Supplementary Fig. 2 were not sufficiently clear and could be misread as implying frequent dual-productive heavy-chain expression, when indeed the figure also included cells with two contigs with identical CDR3 but with constant region isoforms. We have therefore revised the text and Supplementary material to make this distinction explicit. Specifically, we (i) rewrote the Results text around lines 180-186 to clarify that multi-IGH observations in memory B cells are dominated by IGHM/IGHD isoforms sharing an identical V(D)J junction (identical CDR3 nucleotide sequence), consistent with unswitched memory or naive-like phenotypes rather than two independent productive receptors; (ii) updated Supplementary Fig. 2 to include total cell numbers per group (panel a) and absolute cell counts throughout; and (iii) added an alluvial plot in Supplementary Fig. 2b that reports absolute counts and separates cells with multiple productive IGH contigs with identical V region (same VDJ/CDR3) from truly distinct IGH rearrangements (different VDJ/CDR3), as well as separating cells with multiple IGH rearrangements where only one is productive. This indicates that only ~2% of memory B cells in Dataset III contain two distinct IGH rearrangements, whereas the large majority of productive multi-IGH cases correspond to IGHM/IGHD isoforms from the same rearrangement. We also updated the Discussion to reflect a conservative interpretation of multi-contig reconstructions and to avoid over-interpreting secondary/low-support contigs

as evidence of co-expression of multiple functional receptors (lines 279–289). Finally, we note that true dual-productive IGH expression has been reported to be uncommon, and our revised presentation and conservative interpretation are aligned with how multi-contig barcodes are typically handled in single-cell BCR reconstruction workflows.

2. Regarding the datasets presented in Table 1, there were more cells in the original datasets and and publications that investigated them than the cell numbers shown in Table 1. Why were some cells excluded and what was the rationale behind the filtering approach that was employed? This has to be stated in the manuscript.

Response 2.2: We agree and have now stated the inclusion/exclusion rationale explicitly in the manuscript. We added a new Methods subsection, "Benchmark dataset selection and inclusion criteria", describing for each benchmark dataset why the analyses use a subset of the originally generated cells (Methods, lines 384–395), and we also added Table 1 footnotes accordingly. Briefly:

- *Dataset I* includes both paired-end and single-end cells; we analyzed the paired-end subset (n=174) and excluded the single-end subset (n=17) to avoid confounding by sequencing mode.
- *Dataset II (E-MTAB-4825):* consistent with prior validation in the BraCeR study (which analyzed batch 1), we focused on batch 1 and used the cells with available raw FASTQs to ensure that the benchmark is fully reproducible from public data.
- *Dataset III:* we randomly sampled 200 naive and 200 memory B cells (1:1) to construct a tractable panel representing sparse Ig transcript settings; the barcode list is provided in Source Data.
- *Dataset IV:* we included all Seurat-annotated B cells.

3. By looking at the new Figure 3, it is clear that GATHER shows very similar performance to other published tools regarding constant-region assembly. This should be mentioned in the discussion.

Response 2.3: We agree. We have revised the Discussion to state explicitly that, in our ground-truth validation, constant-region (including isotype-level) assignment was comparable to other methods when constant-region sequence was sufficiently covered (Discussion, lines 302–305). We further clarify that GATHeR's main advantage in such settings is improved contiguity and extended constant-region reconstruction, enabling subclass/allele calls and isoform resolution.
